# JAG1 Intracellular Domain Enhances AR Expression and Signaling and Promotes Stem-like Properties in Prostate Cancer Cells

**DOI:** 10.3390/cancers14225714

**Published:** 2022-11-21

**Authors:** Tuyen Thanh Tran, Keesook Lee

**Affiliations:** Laboratory of Developmental Genetics, School of Biological Sciences and Technology, Chonnam National University, Gwangju 61186, Republic of Korea

**Keywords:** JAG1 intracellular domain, androgen receptor, AR splicing variants, androgen-independent activity, prostate cancer stem-like cells

## Abstract

**Simple Summary:**

The expression of androgen receptor variants (AR-Vs) is associated with the development of advanced castration-resistant prostate cancers (CRPCs), while prostate cancer stem cells (PCSCs) have been evaluated as the most dangerous malignant seeding cells. In this study, we found that the overexpression of the processed intracellular domain of JAG1 (JICD) increased the expression of AR-Vs in prostate cancer (PC) cells and enhanced the transactivation of ARs in an androgen-independent as well as androgen-dependent manner. In addition, JICD increased the expression of CSC markers, such as CD133, and altered the expression of components in PCSC-related signaling pathways. These results, together with the increase in cell mobility and stimulation of tumorigenesis by JICD overexpression, suggest a crucial role of JICD in enhancing androgen independence and promoting stem-like properties in PC cells, driving PC cells to be AR positive and CD133^high^ with high self-renewal and survival ability.

**Abstract:**

JAG1 expression is upregulated in high-grade metastatic prostate carcinomas and associated with poor disease-free survival of patients with prostate cancer. Intriguingly, all JAG1-positive prostate carcinomas express JICD although JICD function in prostate cancer (PC) cells is poorly understood. In this study, we found that JICD overexpression increased the expression levels of AR, especially AR-Vs, in PC cell lines and significantly enhanced androgen-independent and androgen-dependent function of ARs. Interestingly, JICD overexpression upregulated the expression of the PCSC marker CD133 in PC cells as the expression of self-renewal markers; namely, NANOG and OCT3/4 increased. In addition, JICD overexpression highly increased the expression of anti-apoptotic BCL-XL protein, while it little affected the expression of apoptotic BIM protein. In 3D cell culture assays, the spheres formed by JICD-overexpressing PC subline cells (C4-2 and CWR22Rv1) were larger than those formed by control (EV) subline cells with undifferentiated morphology. Although JICD overexpression caused quiescence in cell proliferation, it activated the expression of components in PCSC-related signaling pathways, increased PC cell mobility, and promoted in vivo xenograft mouse tumorigenesis. Therefore, JICD may play a crucial role in enhancing androgen independence and promoting stem-like properties in PC cells and should be considered a novel target for CRPC and PCSC diagnostic therapy.

## 1. Introduction

Prostate cancer (PC) is one of the leading lethal malignancies in males, and its development involves androgen receptor (AR) signaling [1,2,3]. AR is composed of four major domains: NH_2_-terminal transactivation domain (NTD), DNA-binding domain (DBD), hinge region, and ligand-binding domain (LBD). The increased expression of constitutively active AR variants (AR-Vs) [4], such as AR-V7 that is generated by splicing between exon 3 and cryptic exon 3 (CE3) and lacking LBD, is associated with the development of advanced CRPC, leading to hormone therapy failure [5,6,7].

Human cancers harbor subsets of cancer cells known as cancer stem-like cells (CSCs), which are slow-cycling (dormant) cells, resting at the G0 cell cycle phase, and staying as quiescent CSC populations [8]. PC stem-like cells (PCSCs) have been evaluated as the most dangerous malignant seeding cells and believed to be a major factor contributing to drug resistance, metastasis, tumor initiation and recurrence, and cancer-related death [9]. Among stem-like markers, CD133 has been considered the best marker of PCSCs that exhibit high colony and sphere formation capacity [10]. OCT3/4 and NANOG are known as transcription factors that regulate the pluripotency and self-renewal of CSCs and stem cells; they have been identified in somatic tumors, including PC [11]. In addition, the apoptotic signaling pathway is downregulated in CSCs, while the expression of anti-apoptotic molecules is high [12]. The population of PCSCs from human PC tumors possess significant self-renewal capacity and express CSC markers, including CD133 [13,14]. Intriguingly, prostaspheres that propagate from human PC tumors have CD133 and stem cell markers, such as OCT4, NANOG, and JAG1 [15]. In addition, spheres formed from CD133+/CD44+ DU145 PCSCs highly express Notch signaling components including JAG1 as well as stem cell markers [16].

JAG1 is a ligand of Notch receptor that plays a critical role in cell-fate determination and regulation of CSC maintenance [17,18]. JAG1 has three different domains: JAG1-extracellular domain JECD, JCTF intermediate, and intracellular domain JICD. JAG1 expression is upregulated in high-grade and metastatic prostate carcinomas and associated with poor disease-free survival of patients with PC [19,20]. In prostate tumors, JAG1 level is high within the prostate tumor-reactive stroma microenvironment that promotes angiogenesis, invasion, and tumorigenesis [21,22,23,24]. All JAG1-positive prostate carcinoma tissues express cytoplasmic JICD [20] although JICD function in PC cells is largely unknown. JICD is located at amino acids 1087–1220 in the full-length JAG1 and serves as a PDZ-ligand (PDZL) for PDZ proteins (PDZP), which play an important role in signaling trafficking, cell–cell communication, signal transduction, and establishment and maintenance of cell polarity and morphology [25,26,27].

The present study demonstrates that JICD enhances AR-V expression and androgen-independent AR activity in PC cells. Intriguingly, JICD overexpression increases PC stem-like cell properties. These results, together with the increase in cell mobility and stimulation of tumorigenesis by JICD overexpression, suggest that JICD may enhance androgen independence and promote stem-like properties in PC cells, thereby driving CRPC progression.

## 2. Materials and Methods

### 2.1. Cell Lines

LNCaP (CRL-1740), HEK 293T (CRL-11268), PC-3 (CRL-1435), and DU145 (HTB-81) cells (American Type Culture Collection, Manassa, VA, USA) were authenticated by STR analysis. PPC-1 (Cellosaurus ID: CVCL_4778) is a clonal derivative of PC-3 (Cellosaurus ID: CVCL_0035) cells and were chromosomally authenticated by STR analysis [28]. C4-2 and CWR22Rv1 cells (Dr. Leland W. Chung’s laboratory at Cedars-Sinai Medical Center, Los Angeles, CA, USA) were characterized via in vitro characterization techniques [29,30]. Recombinant adenovirus E1-expressing HEK-293 (AD-293; 240085) cells were purchased from Agilent Technologies, Inc. (Santa Clara, CA 95051, USA). Cell culture procedures were previously described in detail [31].

### 2.2. Reagents and Plasmids

G418 disulfate salt (Geneticin; A1720) and 5*α*-dihydrotestosterone (DHT) were purchased from Sigma-Aldrich (St. Louis, MO, USA) and Sigma (Poole, UK), respectively.

pARE2-TATA-Luc, GAL4.AR-LBD658-919, VP16.AR1-660, 5xGAL4-luc3, pcR3.1 SRC-1, pcDNA3.AR, pcDNA3.AR-NTD-DBD, and pcDNA3.AR-V7 were previously described [31,32,33,34,35]. HA-JAGGED1 (JAG1) construct, originally generated in Dr. Gerry Weinmaster’s Laboratory (UCLA, Los Angeles, CA, USA) [36], was kindly gifted by Dr. Hee-Sae Park (Chonnam National University, Gwangju, Republic of Korea). pcDNA3.FLAG-JICD was constructed by inserting a human JICD PCR fragment into the pcDNA3.FLAG expression vector within *Eco*RI and *Eco*RV sites. The PCR primers of human JICD are listed in Table S1.

### 2.3. Cell Transfection and Reporter Assays

Cells were transiently transfected with expression constructs or siRNAs and a luciferase reporter construct with pCMV-LacZ or pRSV-LacZ (Clontech) by using Lipofectamine^TM^ 2000 reagent (11668-019; Invitrogen, Carlsbad, CA, USA) in accordance with the manufacturer’s instruction with minor modifications. The transfected cells were starved in media containing 5% cFBS for 24 h and then stimulated with androgen. They were lysed in luciferase lysis buffer (0.2 M Tris-Cl [pH 8.0], 0.2% Triton X100, and 1% NP-40) at 25 °C for 15 min. Luciferase activity was then analyzed in Beetle Luciferin (E1603; Promega Co., Madison, WI, USA) by using a Centro XS3 LB960 luminometer (Berthold Technologies GmbH & Co. KG, 75,323 Bad Wildbad, Germany) and normalized to β-galactosidase activity read by a Versa Max microplate reader (Molecular Devices, LLC., San Jose, CA, USA). The duplet siRNA sequences for the silencing study are listed in Table S1.

### 2.4. RNA Isolation, RT-PCR and qPCR Analysis

Total RNA was isolated from cells by using TRI reagent^®^ [TR 118; Molecular Research Center (MRC), Inc. Cincinnati, OH, USA]. Reverse transcription was performed using Oligo d(T)_15_ (EBT-1523; ELPiS, Taejeon, Republic of Korea) and M-MLV reverse transcriptase kit (M1705; Promega, Madison, WI, USA). mRNA levels were analyzed via RT-PCR by using *Taq* polymerase and quantified by qPCR based on DNA binding of SYBR Green using TOPrealTM qPCR 2X preMIX (SYBR Green with high ROX) (RT501M; Enzynomics, Daejeon, Republic of Korea). Reactions were run on the 96-well StepOnePlus™ Real-Time PCR System (StepOnePlus™ 4376598; Applied Biosystems by Thermo Fisher Scientific Inc., 01620 Vantaa, Finland) and results were imported into Microsoft Excel for further analysis. The relative mRNA expression was calculated as previous described [37]. The primer sequences for gene expression analysis are listed in Table S1.

### 2.5. Western Blot Analysis

Western blot assays were performed as previously described [35]. Proteins were separated through SDS-PAGE and then transferred onto a nitrocellulose blotting membrane (10600004; Amersham^TM^ Portran^TM^ Preminum 0.2 µM; GE healthcare, Little Chalfont, UK). The membrane was blocked and incubated with primary antibodies at 4 °C overnight: anti-JAG1 (sc-8303, Santa Cruz, CA, USA), anti-AR-V7 (Cell Signaling, Danvers, MA, USA), anti-AR (PG21, Millipore, Burlington, MA, USA), anti-AR (441, Santa Cruz, CA, USA), anti-CD133 (Cell Signaling, Danvers, MA, USA), anti-NANOG (Abcam), anti-OCT3/4 (Santa Cruz, CA, USA), anti-Vimentin (Santa Cruz, CA, USA), anti-Ecadherin (Cell Signaling, Danvers, MA, USA), anti-BCl-XL (Epitomics, Burlingame, CA, USA), anti-BIM (Epitomics, Burlingame, CA, USA), anti-FLAG (Sigma), and anti-GAPDH (Santa Cruz, CA, USA). They were then incubated with secondary antibodies for 1 h: HRP-conjugated Piercer^®^ goat secondary anti-mouse IgG (H+L) and Piercer^®^ goat secondary anti-rabbit IgG (H+L). Band signals were visualized on X-ray films (28906839; Amersham HyperfilmTM ECL; GE Healthcare, Little Chalfont, UK) with an ECL^TM^ Western blotting analysis system (RPN2109; GE Healthcare, Little Chalfont, UK). The antibodies are listed in Table S2. The protein band signal was scanned using Image Studio Lite Ver 5.2 (LI-COR, Inc., Lincoln, NE, USA). The relative band intensity of each protein was normalized to GAPDH.

### 2.6. Next-Generation Sequencing (NGS) High-Throughput RNA-Seq Analysis

CWR22Rv1 cells were infected with AdJICD or AdCtrl, maintained for 24 h, and harvested for RNA isolation by using Trizol reagent (Invitrogen). Two independent whole transcriptome analyses (Ebiogen Inc., Seoul, Republic of Korea) were performed as follows. In brief, RNA quality was assessed using an Agilent 2100 bioanalyzer (Agilent Technologies, Amstelveen, The Netherlands), and mRNA was isolated using a Poly(A) RNA selection kit (LEXOGEN, Inc., Vienna, Austria). Libraries were prepared, and high-throughput sequencing was performed as paired-end 100 sequencing in NovaSeq 6000 (Illumina, Inc., San Diego, CA, USA). Fragments per kb per million reads (FPKM) were estimated using Cufflinks (Roberts et al., 2011), and the read count (R) data were processed using the FPKM+Geometric normalization method with EdgeR within R (R development Core Team, Vienna, Austria, 2020). Data mining and graphic visualization were performed using Excel-based Differentially Expressed Gene (ExDEGA) v4.0.3 (Ebiogen Inc., Seoul, Republic of Korea).

Differentially expressed genes (upregulated and downregulated genes) were used for pathway analysis. KEGG pathway and gene ontology (GO) analyses were performed using Shiny version 0.76.3 of the KEGG, curated Reactome, and Immune MSigDB pathway databases provided by Ge Lab of the Bioinformatics Research Group, Department of Mathematics and Statistics, South Dakota State University (http://bioinformatics.sdstate.edu/go/ (accessed on 8 August 2022). Pathway and gene set enrichment analyses were performed with a preranked list of differentially expressed (>2.0-fold) genes between AdJICD- and AdCtrl-infected CWR22Rv1 cells. The genes were clustered, and differences in gene expression were classified using the MeV program.

### 2.7. Generation of JICD-Overexpressing Adenovirus (AdJICD)

JICD-expressing adenoviral construct was generated as previously described [38]. FLAG-JICD fragment was excised from pcDNA3.FLAG-JICD by using *Kpn*I and *Xho*I. Then, it was inserted into pAdTrack.CMV within *Kpn*I and *Xho*I sites to generate pAdTrack.CMV.FLAG-JICD. Purified *Pme*I-linearized pAdTrack.CMV.FLAG-JICD was introduced into BJ5183 bacterial cells harboring the supercoiled backbone vector (AdEasy-1 cells) to generate stable homologous recombinant adenovirus. The recombinant adenoviral construct was screened via *Pac*I digestion and transiently transfected into recombinant adenovirus E1-expressing HEK-293 (AD-293) cells (Agilent Technologies, Inc., Santa Clara, CA 95051, USA) to generate the first virus generation (AdJICD). Viruses were further amplified and purified for infection experiments.

### 2.8. Development of Stable JICD-Expressing CRPC (CWR22Rv1 and C4-2) Cells

C4-2 and CWR22Rv1 cells were transiently transfected with the empty vector pcDNA3.1.FLAG (EV) or pcDNA3.1.FLAG-JICD (JICD), which contains a neomycine-resistant gene; they were then selected using 500 μg/mL G418 (Geneticin; Invitrogen, Carlsbad, CA, USA) for 2 weeks. The clones were picked, expanded, and analyzed for the mRNA and protein expression of JICD. Stable control (EV) and JICD-overexpressing sublines were established and used for in vitro activity and function tests and for in vivo tumorigenesis study.

### 2.9. Cell Proliferation Assays

For cell proliferation, JICD-overexpressing PC subline cells (C4-2 and CWR22Rv1) were seeded on 96-well cell culture plates for 3 days. The medium was replaced each day with fresh media containing inducers. The percent cell growth and cell numbers were obtained using cell counting and MTS assays and measured using a microplate reader system at λ = 490 nm.

For cell viability assays, JICD-overexpressing PC subline cells (C4-2 and CWR22Rv1) were seeded on a 35 mm × 10 mm Corning cell culture dish (10^4^ cells per dish) for 1–6 days and then subjected to crystal violet staining. Three random fields of 0.5% crystal violet-stained cells were imaged via ZEISS microscopy at 10× or 20× magnification.

### 2.10. Colony Formation Assay

Stable EV and JICD-overexpressing PC subline cells (C4-2 and CWR22Rv1) were seeded on a six-well culture plate with 500 cells/well. They were fed with fresh media every 2 days, maintained for 2 weeks, and processed for 0.5% crystal violet-cell staining. Colonies were imaged using a normal camera.

### 2.11. Cell Mobility Assays

For scratch wound-closure assays, JICD-overexpressing PC subline cells (C4-2 and CWR22Rv1) were seeded in 12-well plates (16 × 10^4^ cells per well). After 12 h of seeding, scratch wounds were generated (day 0, D0). The floating cells were removed by fresh medium replacement. The scratched spaces were monitored and imaged daily using EVOS^®^ FL Cell Imaging System (Thermo Fisher Scientific, Waltham, MA 02451, USA) at 4× magnification.

For cell invasion and Boyden Chamber migration assays, JICD-overexpressing PC subline cells (C4-2 and CWR22Rv1) were seeded onto a 24-well Costar chamber comprising an 8 µm polycarbonate membrane, which was precoated with and without 20% phenol red-free Matrix gel (8 × 10^4^ cells per well) for invasion and Boyden Chamber migration assays, respectively. The cells were allowed to grow and invade for 48 h or migrate for 24 h. They were then stained with 0.5% crystal violet. The cells on the inner side of the chamber were gently removed by scraping with a cotton swab and rinsed several times with PBS to remove the excess dye. Three random fields of stained cells were imaged via ZEISS microscopy at 10× magnification.

### 2.12. Three-Dimensional Cell Culture Assays

For sphere formation assay, an ultralow/non-attachment Petri dish (60 mm × 15 mm) was seeded with 4000 cells of stable EV and JICD-overexpressing PC cells (C4-2 and CWR22Rv1). Both attached and non-attached tumor spheres (TSs) were maintained for 7 days. Fresh media were added every 2 days. TSs were collected and analyzed for the expression levels of JICD, AR-Vs, and PC stemness-related markers.

A modified 3D cell culture was prepared with Matrigel^®^ Matrix Phenol Red-free (#356231, Corning, Glendale, AZ, USA). Stable EV and JICD-overexpressing PC C4-2 and CWR22Rv1 cell suspensions containing 100 cells were mixed at 1:1 with cold Matrigel. Cell droplets were generated by pipetting 20 μL of droplets onto a 60 mm × 15 mm ultralow attachment Petri dish. The cell droplets were then incubated in a 5% CO_2_ incubator at 37 °C for at least 1 h to allow the Matrigel to solidify. Complete media (5 mL) were added to cover all droplets, which were maintained for 10 days. Fresh media were added every 2 days.

Spheres were imaged by using EVOS^®^ FL Cell Imaging System (Thermo Fisher Scientific, Waltham, MA 02451, USA) at 10× and 20× magnification.

### 2.13. Xenograft Animal Model

Healthy and microbiologically monitored 4-week-old male NOD.CB17-Prkdc^SCID/J^ mice obtained from Korea Research Institute of Bioscience and Biotechnology (Daejeon, Korea) were gently anesthetized with 40–50 µL of sevofran solution (Hana Pharm Co., Ltd., Gyeonggi-do, Republic of Korea). The stable JICD-overexpressing and EV CWR22Rv1 subline cells (10^7^ cells/site mixed 1:1 with Matrigel) were subcutaneously injected (s.c.) into the shoulder of each mouse. Tumor size and volume were monitored and measured two times a week. Two days after the last tumor measurement, the mice were subjected to CO_2_-induced euthanasia and sacrificed; then, their tumors were extracted and weighed. Animal experiments were performed using six mice in each group. Statistical significance was calculated using two-tailed *t*-test analysis.

Animal care and maintaining conditions were previously described [31]. All animal experiments were approved by the Institutional Animal Care and Use Committee of Chonnam National University (permit number: 2021-17) and have been performed in accordance with the ARRIVE/NC3R guidelines.

### 2.14. Quantification and Statistical Analysis

Data, which were obtained from more than three independent experiments, are presented as the mean ± SEM. Statistical significance was calculated using one-way ANOVA with Tukey’s post hoc test and two-tailed *t*-test analysis.

## 3. Results

### 3.1. JAG1 Regulates the Expression of AR and AR-Vs in PC Cells

To investigate the oncogenic function of JAG1 in PC, we analyzed the expression of JAG1 in PC cell lines. Western blotting analysis revealed that JAG1 protein expression was detected in all tested PC cell lines, namely, AR-positive (LNCaP, C4-2, and CWR22Rv1) and AR-negative (PC-3) cell lines, but only after DHT treatment (Figure S1). The basal level of JAG1 protein was relatively high in LNCaP cells, while its processed JICD protein level was high in LNCaP and PC-3 cells compared with other cell lines. Interestingly, the expression of JAG1 protein and JICD was highly induced by DHT in C4-2 cells but less induced in other PC cells (Figure S1).

**Figure 1:**
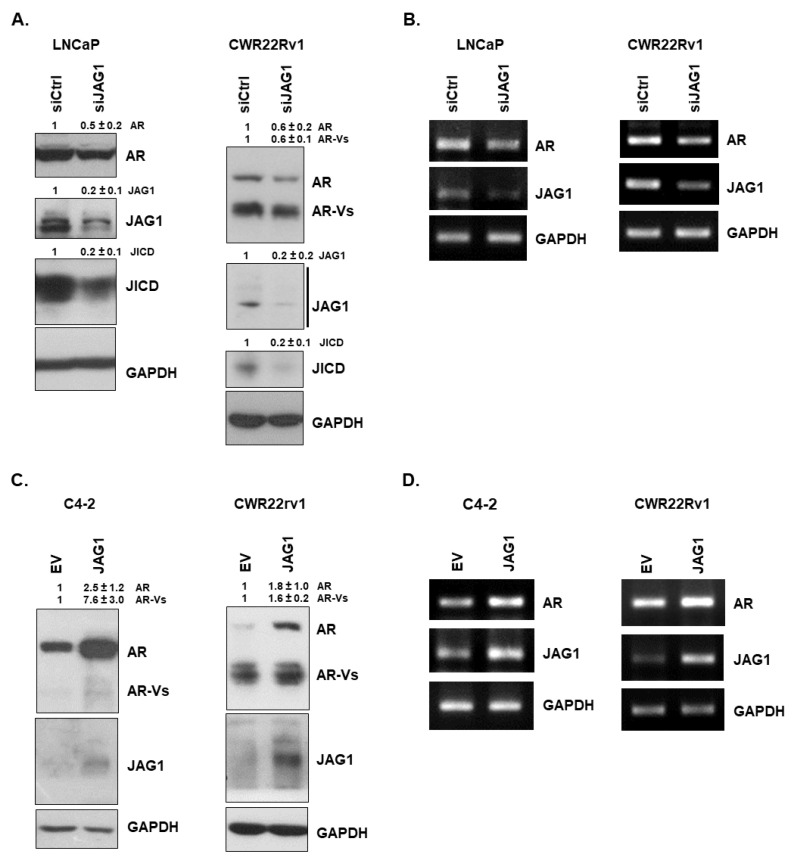
JAG1 regulates the expression of AR and AR–Vs. (**A**,**B**) Silencing of JAG1 decreases the expression of ARs. (**A**) Representative Western blot analysis showing the protein levels of AR and AR–Vs in siCtrl– or siJAG1–transfected androgen–dependent LNCaP and androgen-independent CWR22Rv1 cells. AR and AR–V proteins were detected using rabbit anti–AR (06–680, Millipore). (**B**) RT–PCR analysis showing the mRNA level of AR in siCtrl– or siJAG1–transfected LNCaP and CWR22Rv1 cells. RT–PCR analysis shows a decrease in total AR mRNAs, which contain exon 1–exon 2 (AR), in LNCaP and CWR22Rv1 cells when JAG1 was silenced. (**C**,**D**) Overexpression of JAG1 increases the expression of ARs. (**C**) Representative Western blot analysis showing the protein levels of AR and AR–Vs in EV– or JAG1–transfected C4–2 and CWR22Rv1 cells. AR and AR–V proteins were detected using rabbit anti–AR (06–680, Millipore). (**D**) RT–PCR analysis showing the level of AR mRNAs, which contain exon 3–exon 4 (AR), in EV or JAG1–transfected C4–2 and CWR22Rv1 cells. GAPDH was used as a loading control. Values above Western blots indicate the relative band intensity of each protein normalized to GAPDH and are shown as mean ± SD, which were obtained by densitometrical quantification of at least three independent experiments. All original uncropped Western blot and gel images are shown in Figure S7.

As JAG1 and JICD expression levels are upregulated in 98% of prostate tumors [20], we investigated whether JAG1 regulated AR expression. JAG1 silencing resulted in a decrease in the mRNA and protein expression levels of AR and AR-Vs in LNCaP and CWR22Rv1 cells (Figure 1A,B). Conversely, the overexpression of JAG1 enhanced the expression of AR and AR-Vs at mRNA and protein levels in advanced C4-2 and CWR22Rv1 cells (Figure 1C,D). Collectively, these results suggest the regulatory role of JAG1 in AR gene expression.

### 3.2. JICD Upregulates the Expression of AR-Vs in PC Cells

**Figure 2:**
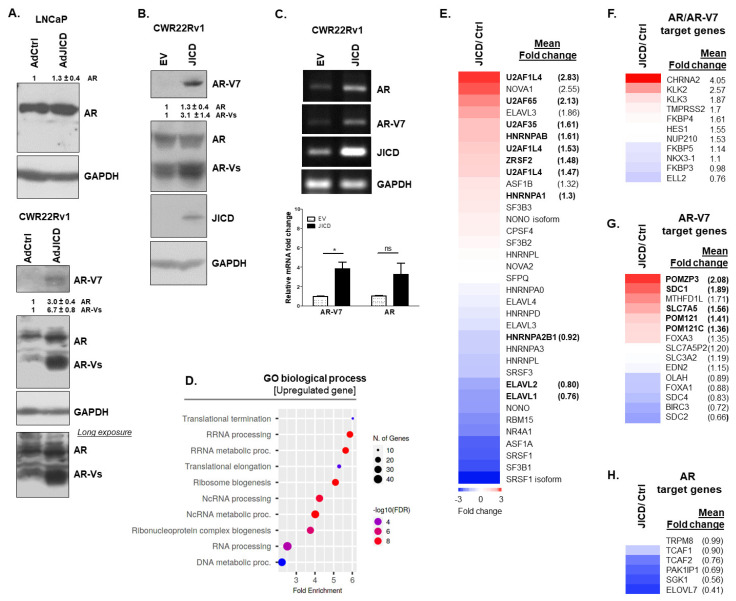
Processed intracellular domain of JAG1 (JICD) increases the expression of AR-Vs. (**A**) Representative Western blot analysis showing the increased protein levels of AR and AR–Vs in AdJICD–infected LNCaP and CWR22Rv1 cells compared to AdCtrl–infected cells. The proteins of AR and ARs including AR–V7 were detected using mouse anti-AR (sc–7305, Santa Cruz) and rabbit anti-AR–V7 (68492, Cell Signaling, Danvers, MA, USA) antibodies. (**B**,**C**) Stable JICD–overexpressing CWR22Rv1 subline cells show the increased expression of ARs, including AR–V7, at protein (**B**) and mRNA (**C**) levels. Representative RT–PCR (**top**) and quantitative RT–PCR (qPCR; **bottom**) analysis of three independent experiments (**C**) revealing a significant increase of AR–V7 mRNA expression in stable JICD–overexpressing CWR22Rv1 subline cells. GAPDH was used as a loading control. Data are shown as mean ± SEM, three independent experiments. *, *p* < 0.01; ns, not significant; two–tailed *t*-test. (**D**) JICD overexpression enriches the expression of genes involved in RNA processing and metabolic process, RNP complex biogenesis, and DNA metabolic process. (**E**) JICD upregulates the expression of some splicing factors involved in the generation of AR–V7 in CWR22Rv1 cells. (**F**–**H**) JICD overexpression enhances the expression of some shared AR/AR–V7 (**F**) and AR–V7 target genes (**G**) and negatively affects the expression of most AR target genes (**H**). False discovery rate (FDR) < 0.05. Values above Western blots indicate the relative band intensity of each protein normalized to GAPDH and are shown as mean ± SD, which were obtained by densitometrical quantification of at least three independent experiments. All uncropped Western blot and gel original images are shown in Figure S7.

We further examined whether JAG1 affected the expression of AR and AR-Vs through its processed JICD in PC cells because JAG1 and JICD expression levels were coordinated in the regulation of AR expression (Figure 1A). JICD overexpression in androgen-dependent LNCaP and androgen-independent CWR22Rv1 cells infected with AdJICD upregulated the protein levels of AR, especially AR-Vs, including AR-V7, although its effect on AR-FL expression was low (Figure 2A). Consistently, the stable overexpression of JICD in CWR22Rv1 cells enhanced the AR expression and strongly upregulated AR-Vs, such as AR-V7 (Figure 2B,C).

RNA-Seq analysis showed that JICD overexpression upregulated approximately 365 genes, while it downregulated approximately 196 genes. Among upregulated genes, JICD overexpression abundantly upregulated the expression of genes involved in RNA processing and metabolism, RNP complex biogenesis, and DNA metabolism (Figure 2D), which might cause mutations and expression of alternative variants found in CRPCs. For example, the hnRNP AB family enhances the expression of AR-V7 in CRPCs [39]; U2AF65 and AFS/SF2 (ZRSF2) occupy the exonic splicing enhancer located between exon 3 and cryptic exon CE3 in the AR gene to regulate the production of AR-V7 positively [6]. RNA-seq analysis showed that the expression levels of U2AF65, AFS/SF2 (ZRSF2), and hnRNP AB increased by approximately 2.1-, 1.5-, and 1.6-fold, respectively (Figure 2E). In addition, gene set enrichment analysis of published AR/AR-V7 target gene sets, AR-V7 target gene sets, and AR target gene sets [40,41] showed that JICD overexpression caused 1.5-to-4-fold induction of some AR/AR-V7 target genes [41] and 1.4-to-2-fold induction of some AR-V7 target genes [40,41], while it mostly downregulated AR target genes (Figure 2F–H). These results suggest that JICD has a regulatory role in the expression of AR variants in PC cells, resulting in the amplification of androgen-independent signaling pathways in CRPCs.

### 3.3. JICD Enhances Androgen-Dependent and Androgen-Independent Transactivation of ARs

**Figure 3:**
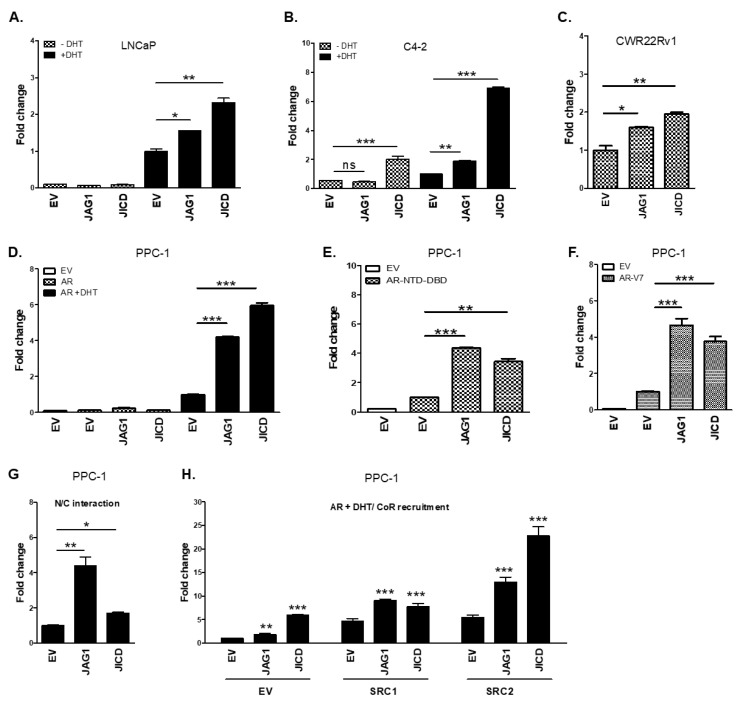
JAG1 and JICD enhance the androgen–dependent and androgen–independent transactivation of ARs. (**A**–**C**) JICD overexpression significantly enhances the transactivation of ARs. Prostate cancer LNCaP (**A**), C4–2 (**B**), and CWR22Rv1 (**C**) cells were transiently transfected with FLAG–JICD or empty vector (EV) along with pARE2–TATA–luc and treated with 1 nM DHT or vehicle. (**D**–**F**) PPC–1 cells overexpressed with AR–FL (**D**), AR–NTD–DBD (**E**), or AR–V7 (**F**) were transiently transfected with FLAG–JICD or empty vector (EV) and pARE2–TATA–luc and treated with 1 nM DHT or vehicle. (**G**) Overexpression of JAG1 and JICD enhances the N/C interaction of AR. PPC1 cells cotransfected with the AR N–terminal (VP16/AR1–660) domain, C–terminal (GAL4/AR–LBD658–919) domain, and 5xGAL4–luc3 reporter, along with JAG1, FLAG–JICD, or empty vector and incubated with 10 nM DHT. (**H**) Overexpression of JAG1 and JICD enhances coactivator recruitment to AR. PPC–1 cells were cotransfected with AR, SRC–1 (NCOA1), or SRC–2 (NCOA2), and JAG1, FLAG–JICD, or empty vector, along with pARE2–TATA–luc and incubated with 1 nM DHT. Luciferase activity was normalized to that of *β*-galactosidase. Data are shown as mean ± SEM. *, *p* < 0.05; **, *p* < 0.01; ***, *p* < 0.001; ns, not significant; one–way ANOVA with Tukey’s post–hoc test.

We further examined the effect of JICD and JAG1 on the transcriptional activity of ARs (full-length AR, AR-FL; AR N-terminal and DBD domain, AR-NTD-DBD; and AR-Vs including AR-V7) via luciferase reporter assays. The overexpression of JAG1 and JICD significantly enhanced the androgen-induced transactivation of endogenous AR in androgen-dependent LNCaP cells and androgen-dependent and -independent C4-2 cells [42]. The androgen-independent transactivation of ARs in C4-2 and CWR22Rv1 cells was also enhanced by JICD and JICD/JAG1 overexpression, respectively (Figure 3A–C). In addition, the overexpression of JAG1 and JICD in PPC-1 cells (AR-negative PC-3-derived prostate cancer cells) markedly enhanced the androgen-dependent and androgen-independent transactivation of exogenous AR-FL, AR-NTD-DBD, and AR-V7 in a dose-dependent manner (Figure 3D–F and Figure S2). Enhancement on AR transactivation by JICD could be further supported by the upregulation of AR/AR-V7 target genes, such as PSA (KLK3), hK2 (KLK2) and TMPRss2, upon JICD overexpression in CWR22Rv1 cells (Figure 2F).

The NH_2_/COOH terminal (N/C) interaction of AR and the recruitment of a coactivator to androgen response elements (AREs) in target gene promoters are important for AR transactivation. JAG1 overexpression showed approximately 4-fold enhancement in the N/C interaction of AR, while JICD overexpression showed approximately 2-fold enhancement (Figure 3G). In addition, the overexpression of JAG1 and JICD further facilitated the recruitment of some known AR coactivators, such as SRC-1 and SRC-2, to AREs, synergistically enhancing AR transactivation (Figure 3H). Further, JICD physically interacted with AR, specifically with the NTD-DBD of AR (Figure S3), suggesting a molecular mechanism through which JICD increased the activity of ARs in androgen-dependent and androgen-independent manners.

### 3.4. JICD Promotes Stem-Like Cell Properties in PC Cells

PCSCs are a subset of cells within prostate tumors; they have a long-term self-renewal potential and cause clinical treatment failure, tumor relapse, and metastasis [43]. Since JICD of JAG1 is involved in malignant cell transformation [44], we investigated whether JICD promotes PC stem-like cell properties in PC cells, leading to advanced PCs. CD133 has been considered the best exemplified and used marker of cancer stem cells (CSCs), including PCSCs [45,46]. Meanwhile, CD44 and CD133 have been used to identify basal prostate stem cells (PSCs) [43] because basal cells in the prostate epithelial layer are CD44^+^p63^+^CK5^+^CK14^+^. Normal PSCs are well known as CD44^+^CD133^+^AR^−/low^PSA^−/low^ [43], while PCSCs are CD133^+^AR^high^ [45].

**Figure 4:**
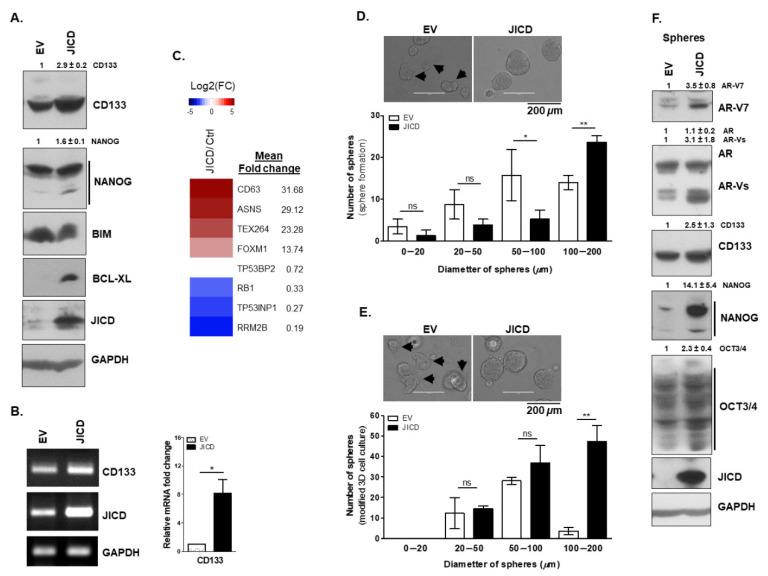
JICD promotes prostate cancer stem–like cell properties in prostate cancer cells. (**A**,**B**) Overexpression of JICD increases CSC marker expression in prostate cancer cells. Representative Western blot analysis (**A**) showing the protein levels of CD133, NANOG, BIM, BCL–XL, and JICD; Representative RT–PCR (**top**) and quantitative RT–PCR (qPCR; **bottom**) analysis of three independent experiments (**B**) revealing the mRNA levels of CD133 and JICD in stable EV– or JICD–overexpressing CWR22Rv1 subline cells. GAPDH was used as a loading control. Data are shown as mean ± SEM, three independent experiments. *, *p* < 0.01; two–tailed *t*-test. (**C**) Heatmap showing the strong upregulation of CD63, ASNS, TEX264, and FOXM1 and the downregulation of RB1 and P53 downstream signaling pathways (RB1, TP53BP2, TP53INP1, and RRM2B), which are related to stem-like cell properties. (**D**,**E**) JICD overexpression facilitates and maintains sphere formation. Representative images of stable EV– or JICD–overexpressing CWR22Rv1 spheres grown for the sphere formation (**D**, **top**) and modified 3D cell culture (**E**, **top**) assays (magnification, ×10 and ×20; bars, 400 and 200 μm, respectively). Arrowheads (**D**,**E**, **top**) indicate the spheres with differentiated morphology. Graphs presenting the number of spheres versus the diameter of spheres (μm) derived from stable EV– or JICD–overexpressing CWR22Rv1 cells, which were grown under sphere formation (**D**, **bottom**) and modified 3D cell culture (**E**, **bottom**) conditions. Data are shown as mean ± SEM. *, *p* < 0.05; **, *p* < 0.01; ns, not significant; one-way ANOVA with Tukey’s post-hoc test. (**F**) Representative Western blot analysis showing that spheres derived from stable JICD–overexpressing CWR22Rv1 cells expressed higher protein levels of CD133, NANOG, OCT3/4, and ARs, especially AR–Vs, including AR–V7, than the control EV cell-derived spheres did. Values above Western blots indicate the relative band intensity of each protein normalized to GAPDH and are shown as mean ± SD, which were obtained by densitometrical quantification of at least three independent experiments. All original uncropped Western blot and gel images are shown in Figure S7.

We first examined the expression of CD133 and the normal stem cell marker NANOG. The stable overexpression of JICD increased the CD133 expression at both mRNA and protein levels in CWR22Rv1 and C4-2 cells (Figure 4A,B and Figure S4A,B). We also observed that the mRNA expression of CD133 was upregulated when PC cells (LNCaP, C4-2, and CWR22Rv1) were infected with AdJICD compared with that infected with AdCtrl (Figure S4C). Furthermore, JICD overexpression in CWR22Rv1 and C4-2 cells increased the protein expression of NANOG (Figure 4A and Figure S4A). Overexpression of JICD in PC-3 and DU145 cells, patient-derived metastatic prostate carcinoma cell lines, also showed an increase in the protein levels of CD133, by approximately ~1.6-fold and 2.8-fold, respectively (Figure S4D). In addition, JICD overexpression highly increased the expression of anti-apoptotic BCL-XL protein in CWR22Rv1 and C4-2 cells, while it little affected the expression of apoptotic BIM protein (Figure 4A and Figure S4A).

Several signaling pathways are involved in PCSC maintenance and tumorigenesis. For example, the activation of PI3K-AKT, RAS-MAP kinase, and JAK-STAT3 signaling pathways promotes the self-renewal activity of PCSC [43]. In the present study, JICD overexpression amplified these signaling pathways (Figure S4E) and possibly enhanced PC stem-like cell properties. CSCs can undergo extensive metabolic reprogramming [47], showing rapid DNA damage repair [48] and enhanced cancer drug resistance [49]. DNA repair proteins play crucial roles in CSC maintenance and adaptation to replication and oxidative stress [50]. Among them, testis-expressed protein 264 (TEX264) [51] and forkhead box M1 (FOXM1) [52] are involved in DNA repair response. In addition, FOXM1 is linked to the enhanced expression of stem cell markers in tumor samples, thereby promoting stem cell properties [53]. Our RNA-Seq analysis showed that JICD overexpression activated DNA repair and cancer drug resistance pathways (Figure S4E). We also observed an abundant upregulation of TEX264 and FOXM1 expression by approximately 23- and 14-fold, respectively (Figure 4C).

Asparagine synthetase (ASNS) is upregulated in and associated with CRPC [54], and the excessive asparagine is considered a potential mechanism that promotes the translation of stem cell regulatory proteins [55]. JICD overexpression abundantly upregulated the expression of ASNS (~29-fold), which may support the role of JICD in enhancing PCSC properties. We also observed a ~32-fold upregulated expression of CD63 (Figure 4C), a diagnostic marker of PC and key component of cancer stem cell-derived extracellular vesicles involved in the maintenance of stemness phenotype [56]. In addition, the expression of retinoblastoma tumor suppressors RB1 and p53, which are downregulated in CRPC and associated with cancer drug resistance [57], significantly decreased upon JICD overexpression; that is, the expression of RB1 decreased by ~3-fold, and the expression of TP53 downstream target genes decreased as followed: RRM2B and variants by ~5.3-fold, TP53BP2 by ~1.4-fold, and TP53INP1 by ~3.7-fold (Figure 4C).

CSC characteristics and consequences in the context of tumor formation and metastasis, including proliferation, senescence, quiescence, cell cycle re-entry, and stemness, are complicated [58]. The forkhead box O (FOXO) signaling pathway plays a pivotal role as a tumor suppressor in many cancers, and is involved in the regulation of malignant stem cells [59]. Several members of the FOXO subfamily are directly phosphorylated and inhibited by PI3K-AKT signaling pathway. The restoration of self-renewal potential in the context of FOXO deficiency with constitutive activation of the PI3K-AKT pathway has been reported in most myeloid leukemias solid tumors. Intriguingly, we observed a similar situation in which JICD overexpression downregulated the expression of genes involved in the FOXO signaling pathway (~6.7-fold) with the activation of PI3K-AKT pathway (Figure S4E,F). In addition, miRNAs regulate the stemness of PCSCs by targeting stemness-related transcription factors (OCT3/4, KLF4, and NANOG), CSC/PCSC markers (CD133 and CD44), epithelial–mesenchymal transition (EMT), metastasis-related factors, and drug resistance-related factors. miRNAs are also associated with the regulation of several stemness-related pathways including PI3K-AKT and MAPK pathways [60]. In the present study, JICD overexpression downregulated miRNA expression by ~6.2-fold (Figure S4F). Cellular senescence is an endogenous tumor suppressor mechanism, and the tumor cells that escaped or released from senescence acquire drug resistance, tumorigenicity, and CSC characteristics [61]. Interestingly, we observed the downregulated expression of senescence genes in JICD-overexpressed cells (Figure S4F).

Although many technologies and assays have been proposed for the identification of CSCs, sphere formation assay and in vivo tumor xenograft immunodeficient mouse models have been considered as the gold standard for CSC identification [62]. Therefore, we performed 3D cell culture assays to further examine the role of JICD in enhancing PC stem-like cell properties. Under non-adherent culture conditions, the spheres formed by the stable JICD-overexpressing PC subline cells (CWR22Rv1 and C4-2) were larger than those formed by the control (EV) subline cells, but the total number of spheres had no significant differences (Figure 4D and Figure S4G). In the modified 3D cell culture using Matrigel, JICD-overexpressing PC subline cells formed a higher number of spheres and retained the ability to form larger spheres than the control (EV) did (Figure 4E and Figure S4H). Furthermore, the control EV cell-derived spheres showed differentiated morphology with brighter and improper round shape and protruded bleds of empty cytoplasm (Figure 4D,E, top, arrowheads) unlike the JICD-overexpressing cell-derived spheres showing compact structure with darker color and well-defined border/boundaries (Figure 4D,E). We also examined the protein expression of some important markers of cancer stem cells and AR-Vs in spheres. Consistent with previous observations, the protein levels of CD133, NANOG, and OCT3/4 and AR-Vs containing AR-V7 in JICD-overexpressing CWR22Rv1 spheres were higher than those in EV spheres (Figure 4F). Collectively, these results suggest the crucial role of JICD in enhancing stem-like cell properties in PC cells.

### 3.5. JICD Increases PC Cell Mobility and In Vivo Tumorigenesis

**Figure 5:**
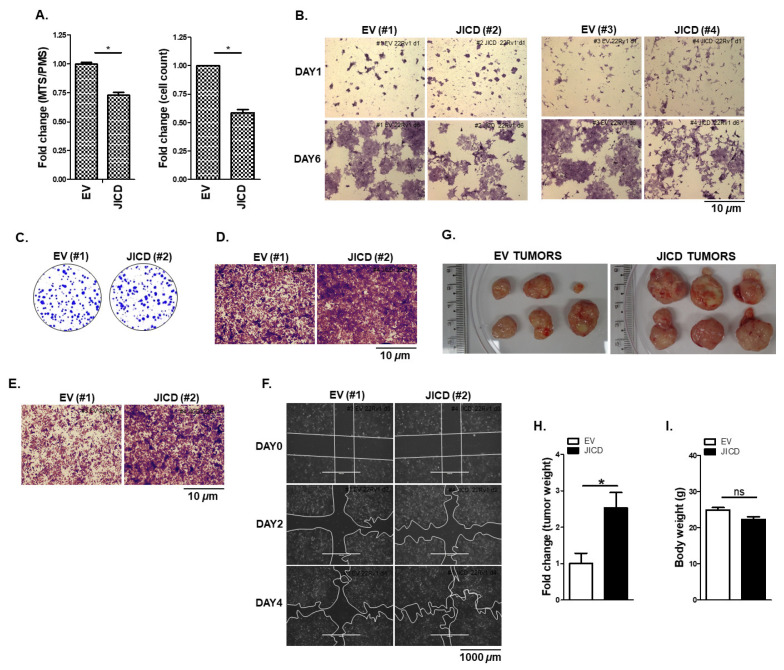
JICD overexpression increases prostate cancer cell mobility and in vivo tumorigenesis. (**A**,**B**) Stable JICD overexpression reduces the proliferation and viability of CWR22Rv1 cells. Stable EV (#1) or JICD (#2) CWR22Rv1 subline cells were maintained for 3 days and then subjected to MTS/PMS assays (**A**, **left**) or cell counting assay (**A**, **right**). Stable EV (#1 and #3) or JICD (#2 and #4) subline cells were maintained for 1 and 6 days, stained with crystal violet, and imaged via ZEISS microscopy at 20× magnification (scale bar 10 μm) (**B**). Data are shown as mean ± SEM. *, *p* < 0.01; two-tailed *t*-test. (**C**) Colony size increases in stable JICD-overexpressing CWR22Rv1 subline cells compared with that in the control without significant difference in the total number of colonies between them. Stable EV (#1) or JICD (#2) subline cells were maintained for 2 weeks and then processed for 0.5% crystal violet staining. Colonies were imaged with a normal camera. (**D**–**F**) JICD increases the mobility of prostate cancer cells. Invasion assays of stable EV (#1) or JICD (#2) CWR22Rv1 subline cells (**D**). Cell invasion was allowed to occur for 48 h, stained with crystal violet, and imaged via ZEISS microscopy at 10× magnification (Scale bar = 10 μm). Boyden Chamber migration assay of stable EV– or JICD–overexpressing CWR22Rv1 cells (**E**). Cells were allowed to migrate for 24 h, stained with crystal violet, and imaged with ZEISS microscopy at 10× magnification (Scale bar = 10 μm). Scratch wound-closure assay of stable EV (#1) or JICD (#2) CWR22Rv1 subline cells (**F**). Scratch wounds were generated (day 0, D0), and wound distances were monitored for 2 (D2) and 4 (D4) days; they were then imaged using EVOS^®^ FL Cell Imaging System at 4× magnification (Scale bar = 1000 μm). Boundary lines were simply drawn using the shape tool from PowerPoint. (**G**–**I**) JICD overexpression promotes CWR22Rv1 xenograft tumor growth in vivo. Stable JICD–overexpressing (JICD; #2) or control (EV; #1) CWR22Rv1 subline cells were implanted into the shoulders of 4–week–old male NOD.CB17–PrkdcSCID/J mice, and tumors were allowed to grow for 7 weeks. Representative tumors (**G**) dissected from mice after 7 weeks. Tumor weight (**H**) and body weight (**I**) measured after 7 weeks. Data are shown as mean ± SEM, two independent biological in vivo experiments (*n* = 6 mice per each group). *, *p* < 0.01; ns, not significant; two–tailed *t*-test.

Considering that JICD affected AR signaling in PC cells, we investigated the effect of JICD on cell proliferation and mobility, which are the downstream cell behaviors of AR signaling. We generated two stable JICD-overexpressing sublines (JICD; sublines #2 and #4) and two control sublines (EV; sublines #1 and #3) of CWR22Rv1 and C4-2. The stable JICD-overexpressing CWR22Rv1 and C4-2 sublines (JICD; sublines #2) expressed JICD more than the other CWR22Rv1 and C4-2 sublines (JICD; sublines #4), respectively (Figure S5A). The stable overexpression of JICD decreased the cell growth of CWR22Rv1 and C4-2 cells (Figure 5A,B and Figure S5B,C) probably because of the decrease in cell cycle signaling (Figure S4C) with an increase in stem-like cell properties upon JICD overexpression (Figure 4 and Figure S4). Since both JICD sublines (sublines #2 and #4) of both CWR22Rv1 and C4-2 cells showed a similar cell growth behavior with decreased cell viability compared to the control EV sublines (Figure 5B and Figure S5C), we used the stable EV (subline #1) and JICD (subline #2) sublines for in vitro and/or in vivo experiments through this study.

Although JICD overexpression decreased the PC cell growth (Figure 5A,B and Figure S5B,C), JICD-overexpressing PC cells gradually formed larger colonies than the control EV cells did (Figure 5C and Figure S5D) and showed the markedly increased ability of cell invasion and migration (Figure 5D–F and Figure S5E–G). These results suggest that JICD enhances PC stem-like cell properties. Thus, cells slowly grow, but they have high survival and invasive capacities as seeding cells for tumor initiation.

We further investigated the in vivo effect of JICD on prostate tumorigenesis by using xenograft mouse models produced with JICD (#2) and EV (#1) CWR22Rv1 subline cells. The average weight of tumors from the JICD mice was approximately 2.5-fold heavier than that of the tumors from the EV mice (Figure 5G,H), but the body weight and other organs had no significant differences (Figure 5I and Figure S5H). Intriguingly, some new small tumors formed from original JICD tumors (Figure 5G, right panel), suggesting the role of JICD in the promotion of micrometastases [63]. Therefore, JICD could be implicated in the invasion, migration, and in vivo tumorigenesis of advanced CRPC cells.

## 4. Discussion

JAG1 has three distinct domains. The domain JECD functions as a specific ligand of Notch. JICD has distinct functions, including the inhibition of Notch signaling [64] and the promotion of Notch1 intracellular domain (NICD) degradation [65]. However, the cellular function of JAG1 signaling via JICD remains largely unknown, although JICD has been shown to repress the expression of steroidogenic genes, resulting in the reduced production of androgens [66]. In this study, we found for the first time that JICD upregulated the expression and androgen-independent activity of AR-Vs in PC cells, suggesting a pivotal role of JICD in the development of some androgen-independent CRPCs.

The expression levels of JAG1 and its active intracellular domain JICD are upregulated in high-grade and metastatic prostate carcinomas and associated with the poor disease-free survival of patients with PC [19,20]. Consistent with these observations, our results showed that JICD overexpression significantly increased cell migration and invasion; enhanced stem-like cell properties by upregulating the expression of stemness, anti-apoptotic, and CSC marker genes; and promoted in vivo tumorigenesis in the xenograft mouse model; however, it decreased PC cell growth. Similarly, a previous study demonstrated that JICD decreases myocyte proliferation as the number of apoptotic cardiomyocytes decreases via the inhibition of canonical Notch signaling [64]. In addition, the overexpression of JAG1 intracellular domain (ICD), DLL1-ICD, DLL4-ICD, and NOTCH1-ICD inhibits endothelial proliferation but not migration and adhesion [67]. These results suggest a novel role of JICD in the development of advanced metastatic and drug-resistant CRPCs by enhancing cell migration and stem-like cell properties in PC cells.

Human estrogen receptor-related receptor gamma (ERRγ) was reported to suppress cell proliferation and tumor growth of androgen-sensitive and androgen-insensitive PC cells [68] and JICD overexpression resulted in a depletion of ERRγ (Figure S6A, ESRRG). In addition, homeobox (HOX) genes such as HOXB7 [69], HOXC8 [70], and HOXB13 [71], which are used as PC diagnostic markers, are involved in malignant cell transformation and/or cancer progression. HOXB13 is also a pivotal upstream regulator of AR-V7-driven transcriptomes that are often cell context-dependent in CRPC [71]. JICD overexpression resulted in an approximate 1.5~1.7-fold increase in the expression of such homeobox (HOX) genes in CWR22Rv1 cells (Figures S4C and S6A). These results suggest a role of JICD in facilitating and promoting the CRPC progression of PC cells.

Many transcription factors (TFs) are involved in the regulation of cell proliferation and self-renewal of stem cells and cancer stem cells. For example, KLF4 serves as a prostate tumor suppressor but plays a role in PCSC homeostasis [72]. Its overexpression inhibits the proliferation and self-renewal of tumor-initiating cells and the malignant transformation of stem cells. Conversely, its downregulation enhances PC stem-like cell properties, resulting in invasive sarcomatoid tumors and increased prostate tumorigenesis [72]. Intriguingly, in our study, JICD overexpression decreased KLF4 expression (Figure S6B), promoting in vitro PC stem-like cell properties and in vivo invasive tumorigenesis in the xenograft mouse model. However, its overexpression did not strongly alter other embryonic stem cell (ESC)-related genes (Figures S4C and S6B). Therefore, JICD likely participates in PCSC homeostasis through a mechanism opposite that of KLF4.RNA-seq analysis, which revealed that JICD overexpression altered the expression of genes involved in immune responses; that is, immune tolerance-related genes were enhanced (Figure S6C), while immune defense-related genes were downregulated (Figure S6D). These results provide additional evidence elaborating a previous report, which showed that the upregulation of JAG1 alters prostate tumor histopathology and microenvironment by promoting reactive stroma formation, resulting in the facilitation of CRPC progression [22].

Analysis of JAG1 expression in human tumor samples from 154 men revealed that JAG1 is more highly expressed in metastatic PC than in localized PC or benign prostatic tissues [19]. In addition, high JAG1 expression is associated with PC recurrence after radical prostatectomy for clinically localized disease, while CSCs have been suggested to be responsible for cancer recurrence [73]. Intriguingly, ~80% of advanced prostate patients showed bone metastasis of prostate tumors [74]. Bone marrows have been suggested to serve niches for the enrichment and homeostasis of stem cells and probable stem-like cells. In consistent with these clinical reports, PC-3 cells, which are derived from metastatic bone of prostate carcinoma and positive for stem cell-associated marker CD44, expressed high levels of JICD (Figure S1). It has been also reported that the expression of JAG1/JICD was significantly upregulated in the CD133high/CD44high DU145 PCSCs compared with non-PCSCs [16]. We also observed an increase of CD133 protein expression when JICD was overexpressed in PC-3 and DU145 cells (Figure S4D). VCaP cells, another metastatic prostate carcinoma-derived cell line, are AR-positive, sensitive to a low level of androgens, and widely used as preclinical CRPC models. Therefore, it will be very interesting to examine the effect of JICD on the expression of stem-like cell markers including CD133 as well as the expression of ARs in VCaP cells. All together with the findings in this study, JICD possibly promotes the progression of PC cells to advanced metastatic CRPCs and enhances the PC stem-like cell properties. Therefore, JICD could be an alternative therapeutic target for the treatment of metastatic CRPCs and PC recurrence.

## 5. Conclusions

JICD increases the androgen independence of AR signaling in PC cells by enhancing both the expression of ARs and androgen-independent AR function. It also promotes tumorigenesis by enhancing the PC stem-like cell properties, migration, and invasion of PC cells. These results suggest that JICD is an important factor of the progression of PC cells to advanced metastatic CRPCs. Therefore, JAG1 signaling and/or its active intracellular domain, JICD, can be considered an alternative therapeutic target for advanced PCs and detection marker of PCSC bulks from patients who experience therapeutic failure or have resistant and recurrent cancer.

## Data Availability

The data that support the findings of this study are contained within the article and available from the corresponding author upon reasonable request.

## Supplementary Materials

The following supporting information can be downloaded at: https://www.mdpi.com/article/10.3390/cancers14225714/s1, Figure S1: JAG1 and JICD are expressed in prostate cancer cell lines; Figure S2: Overexpression of JAG1 and JICD enhances androgen-dependent and androgen-independent AR transactivation in a dose-dependent manner; Figure S3: JICD physically interacts with the NTD-DBDof AR; Figure S4: JICD promotes prostate cancer stem-like cell properties; Figure S5: JICD overexpression in C4-2 cells increases the mobility of prostate cancer cells; Figure S6: JICD overexpression alters the expression of genes facilitating CRPC progression; Figure S7: Original uncropped Western blot and gel images (Supporting Data); Table S1: Oligonucleotides; Table S2: Antibodies and sources [75,76].

## Abbreviations

AR	androgen receptor
ARE	androgen-response element
AR-Vs	androgen receptor variants
AR-FL	full-length AR
AR-NTD-DBD	AR N-terminal and DNA-binding domain
CE3	cryptic exon 3
CRPC	castration-resistant prostate cancer
DBD	DNA-binding domain
LDB	ligand-binding domain
NTD	NH2-terminal transactivation domain
JAG1	JAGGED1
JICD	JAG1 intracellular domain
PCSC	prostate cancer stem cell
