# Supplementary material for: JAG1 Intracellular Domain Enhances AR Expression and Signaling and Promotes Stem-like Properties in Prostate Cancer Cells
Source: Cancers (Basel). 2022 Nov 21;14(22):5714. doi: 10.3390/cancers14225714 (PMC9688638; doi:10.3390/cancers14225714)
Supplement: Supplementary file 1 [file cancers-14-05714-s001.zip › cancers-1927011-supplementary.pdf]

Supplementary Materials

JAG1 intracellular domain enhances AR expression and signaling and promotes stem-like properties in prostate cancer cells

Tuyen Thanh Tran and Keesook Lee*

Laboratory of Developmental Genetics, School of Biological Sciences and Technology, Chonnam National University, Gwangju 61186, Republic of Korea
* Correspondence: klee@chonnam.ac.kr; Tel.: +82-62-530-0509; Fax: +82-62-530-0500

1. Supplementary Materials and Methods

1. Co-Immunoprecipitation (Co-IP)

Co-IP assays were performed as previously described [1,2]. HEK293T cells were coexpressed with AR-FL, AR-NTD-DBD, or AR-V7 and FLAG-JICD for 24 h in the absence or presence of DHT. The cells were then sonicated in 25 mM Tris-Cl (pH 8.0) buffer containing protease inhibitors. The supernatant was collected and processed for Co-IP assays by using protein A/G PLUS-Agarose beads (Santa Cruz Biotechnology, Dallas, Texas, USA) and anti-AR (PG-21) or anti-FLAG antibody. Proteins were then detected through western blot analysis.

2. Supplementary Figures and Supplementary Figure Legends

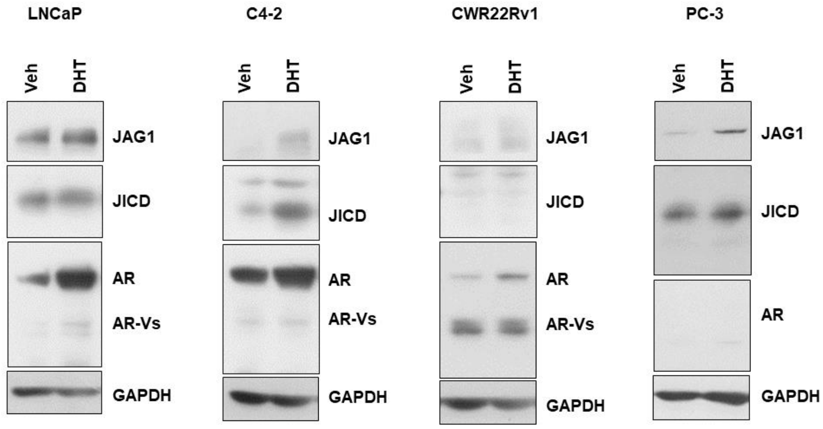

Figure S1. JAG1 and JICD are expressed in prostate cancer cell lines. JAG1 and JICD protein levels are induced by DHT treatment. Representative western blot analysis showing the protein levels of AR, AR-Vs, JAG1, and JICD in prostate cancer cell lines, which were treated with DHT or vehicle for 24 h.

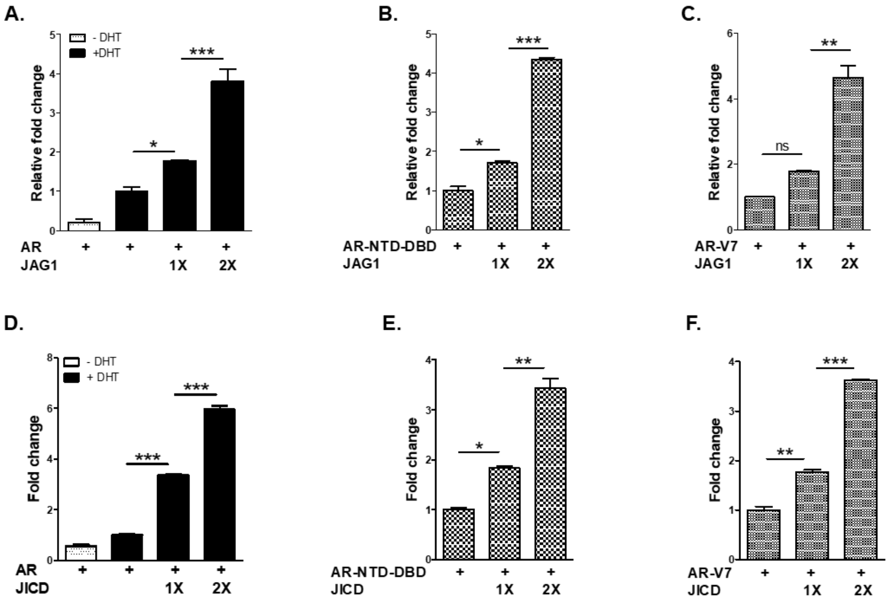

Figure S2. Overexpression of JAG1 and JICD enhances androgen-dependent and androgen-independent AR transactivation in a dose-dependent manner. (**A-C**) JAG1 overexpression enhances the transactivation of exogenous ARs in a dose-dependent manner. PPC-1 cells overexpressed with AR-FL (**A**), AR-NTD-DBD (**B**), or AR-V7 (**C**) were transiently transfected with different amounts of JAG1 and pARE2-TATA-luc and treated with 1 nM DHT or vehicle. (**D-F**) JICD overexpression enhances the transactivation of exogenous ARs in a dose-dependent manner. PPC-1 cells overexpressed with AR-FL (**D**), AR-NTD-DBD (**E**), or AR-V7 (**F**) were transiently transfected with different amounts of FLAG-JICD and pARE2-TATA-luc and treated with 1 nM DHT or vehicle. Luciferase activity was normalized to that of β-galactosidase. Data are shown as mean ± SEM. *, $p < 0.05$; **, $p<0.01$; ***, $p<0.001$; ns, not significant; one-way ANOVA with Tukey's post-hoc test.

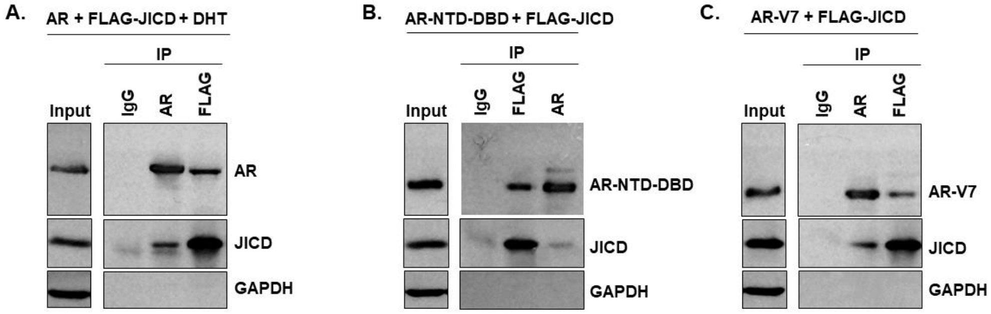

Figure S3. JICD physically interacts with the NTD-DBD of AR. HEK-293T cells overexpressed with AR-FL (**A**), AR-NTD-DBD (**B**), or AR-V7 (**C**) were transiently transfected with FLAG-JICD and treated with 10 nM DHT or vehicle. Physical interaction between JICD and AR-FL (**A**), AR-NTD-DBD (**B**), and AR-V7 (**C**) was examined through co-immunoprecipitation (Co-IP) performed using anti-FLAG or anti-AR antibodies. Proteins were detected via western blot analysis using anti-JAG1 and anti-AR antibodies.

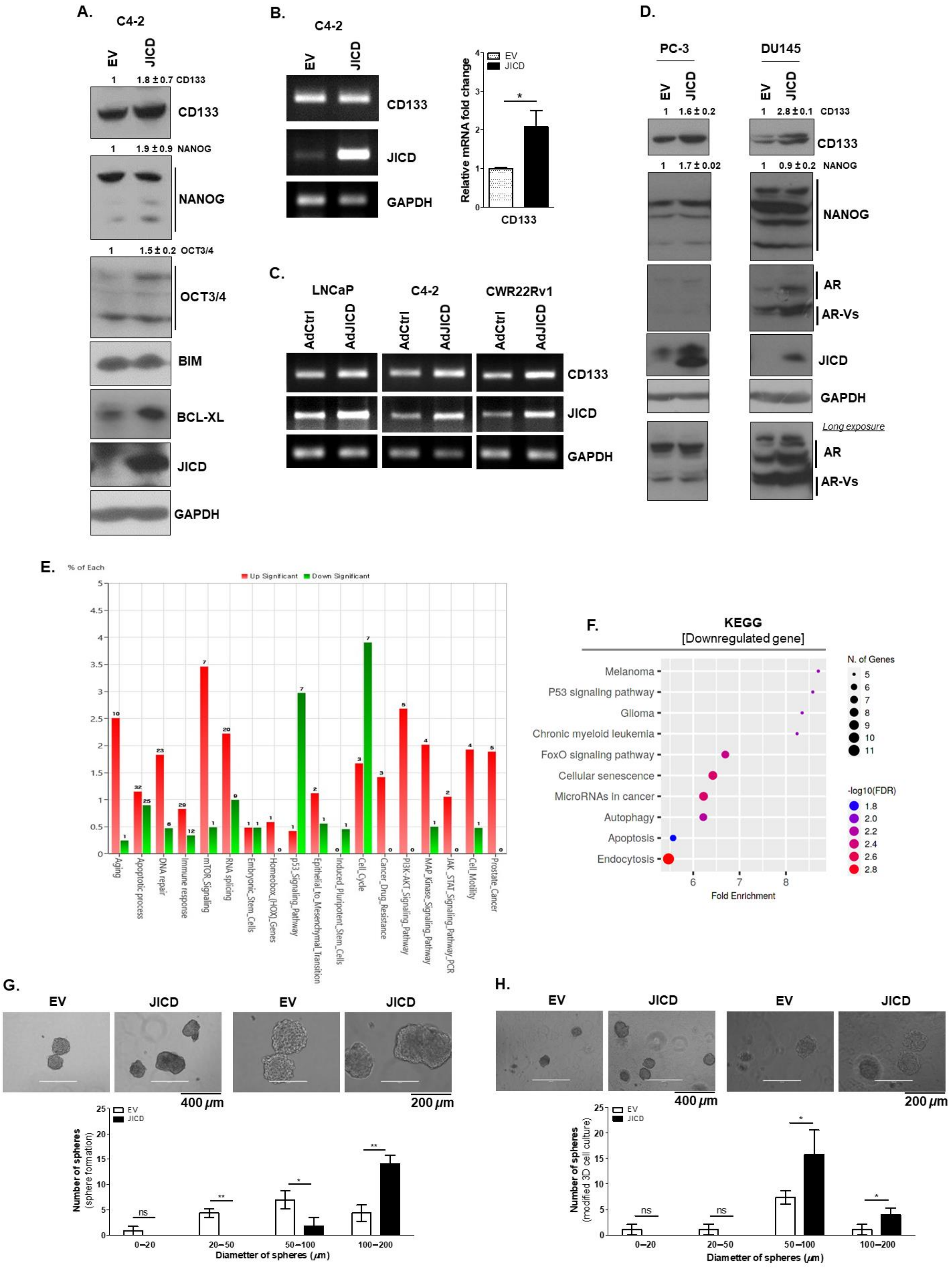

Figure S4. JICD promotes prostate cancer stem-like cell properties. (**A-D**) JICD overexpression increases CSC marker

expression in prostate cancer cells. Representative western blot (**A**) analysis showing the protein levels of CD133, NANOG, BIM, BCL-XL, and JICD in stable EV- or JICD-overexpressing C4-2 subline cells. Representative RT-PCR (**top**) and quantitative RT-PCR (qPCR; **bottom**) analysis of three independent experiments (**B**) showing the mRNA levels of CD133 and JICD in stable EV- or JICD-overexpressing C4-2 subline cells. GAPDH was used as a loading control. Data are shown as mean ± SEM of two independent experiments. *, $p < 0.05$; two-tailed t-test. Representative RT-PCR (**C**) analysis showing the mRNA levels of CD133 and JICD in AdCtrl- or AdJICD-infected prostate cancer cell lines (LNCaP, C4-2, and CWR22Rv1). Representative western blot (**D**) analysis showing the protein levels of CD133, NANOG, AR, and AR-Vs in PC-3 (**left**) and DU-145 (**right**) cells, which were transiently overexpressed with EV or JICD. Proteins were detected using rabbit anti-CD133 (Cell Signaling), rabbit anti-NANOG (Abcam), and anti-AR (Millipore) antibodies. (**E-F**) RNA-seq analysis showing the effect of JICD on the expression of components in signaling pathways related to PCSC development. Gene Ontology (GO) using Excel based Differentially Expressed Gene (ExDEG) analysis revealing the activation of cancer drug resistance, DNA repair, PI3K-AKT, RAS-MAP kinase, and JAK-STAT3 signaling pathways and downregulation of cell cycle and p53 signaling pathways in JICD-overexpressing CWR22Rv1 cells (**E**). KEGG pathway analysis showing the downregulation of genes involved in cancer negative regulatory and tumor suppressor signaling pathways (senescence, apoptosis, microRNAs, and p53 signaling pathway) in JICD-overexpressing CWR22Rv1 cells (**F**). False discovery rate (FDR) < 0.05. (**G-H**) Overexpression of JICD facilitates sphere formation. Representative images of stable EV- or JICD-overexpressing C4-2 spheres grown for sphere formation (**G**) and modified 3D cell culture (**H**) assays (magnification, x10 and x20; bars, 400 and 200 μm; respectively). Graphs showing the number of spheres versus the diameter of spheres (μm) derived from stable EV- or JICD-overexpressing C4-2 cells grown under sphere formation (**G, bottom**) and modified 3D cell culture (**H, bottom**) conditions. Data are shown as mean ± SEM. *, $p < 0.05$; **, $p<0.01$; ns, not significant; one-way ANOVA with Tukey's post-hoc test. Values above western blots indicate the relative band intensity of each protein normalized to GAPDH and are shown as mean ± SD, which were obtained by densitometrical quantification of at least three independent experiments. All original uncropped western blot and gel images are shown in Figure S7.

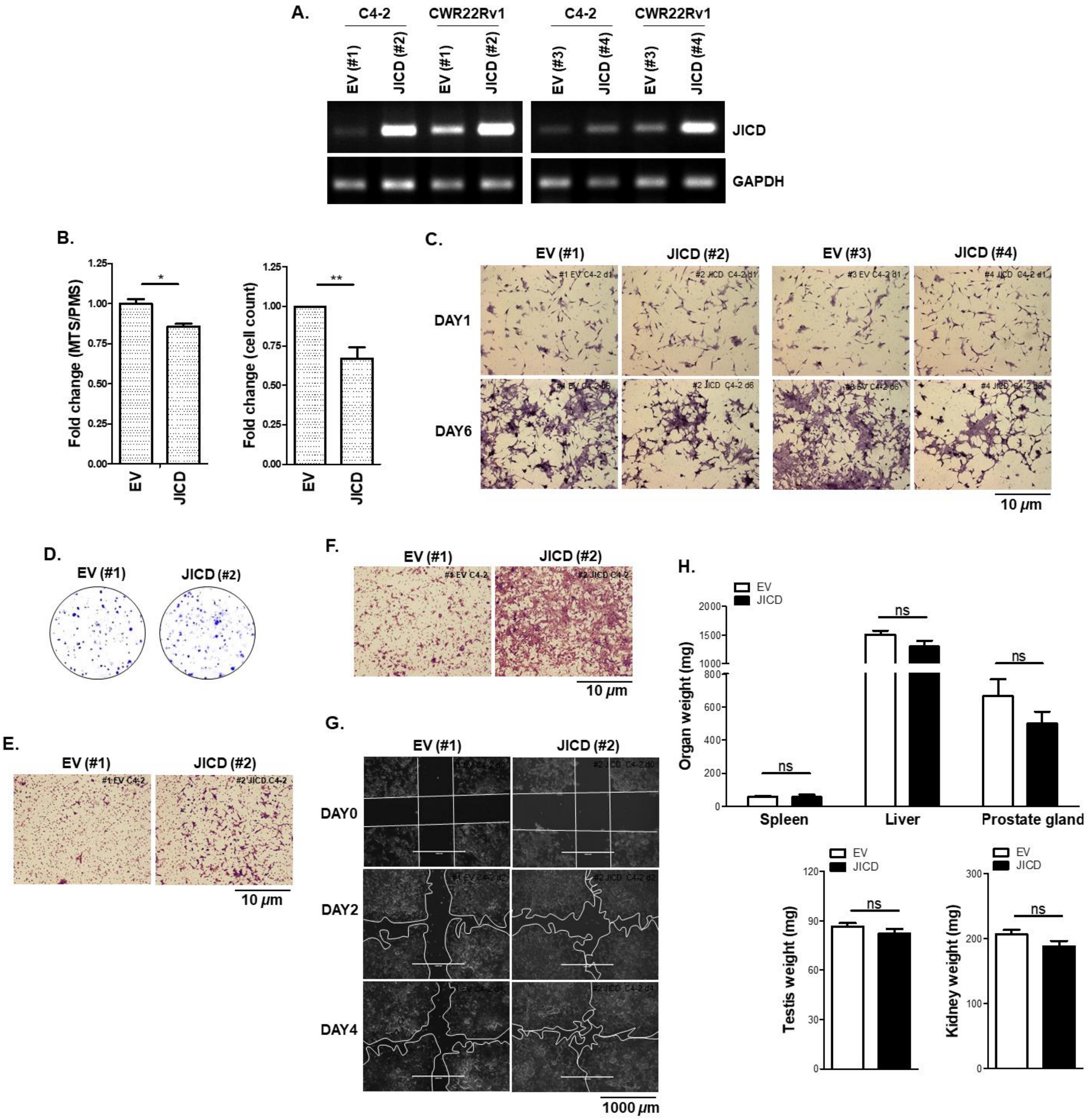

Figure S5. JICD overexpression in C4-2 cells increases the mobility of prostate cancer cells. (**A**) RT-PCR analysis showing JICD expression in the JICD overexpressing C4-2 subline cells (JICD; #2 and #4) and the control subline cells (EV; #1 and #3). (**B-C**) Stable JICD overexpression reduces the proliferation and viability of C4-2 cells. Stable EV- or JICD-overexpressing subline cells were maintained for 3 days and subjected to MTS/PMS assays (**B, left**) or cell counting assay (**B, right**). Data are shown as mean ± SEM. *, $p < 0.05$; **, $p<0.01$; two-tailed t-test. Stable EV (#1 and #3) or JICD (#2 and #4) subline cells were maintained for 1 and 6 days, stained with crystal violet, and imaged via ZEISS microscopy at 20× magnification (Scale bar 10 μm) (**C**). (**D**) The colony size of stable JICD-overexpressing C4-2 cells increases compared with that of the control without significant difference in the total number of colonies numbers between them. Stable EV (#1) or JICD (#2) C4-2 subline cells were maintained for 2 weeks and then processed for 0.5% crystal violet cell staining. Colonies were imaged with a normal camera. (**E-G**) JICD regulates prostate cancer cell

mobility. Invasion assays of stable EV- or JICD-overexpressing C4-2 cells (**E**). Cell invasion was allowed to occur for 48 h, stained with crystal violet, and imaged via ZEISS microscopy at 10× magnification (Scale bar = 10 μm). Boyden Chamber migration assay of stable EV- or JICD-overexpressing C4-2 cells (**F**). Cells were allowed to migrate for 24 h, stained with crystal violet, and imaged via ZEISS microscopy at 10× magnification (Scale bar = 10 μm). Scratch wound-closure assay of stable EV- or JICD-overexpressing C4-2 cells (**G**). Scratch wounds were generated (day 0, D0), and wound distances were monitored for 2 (D2) and 4 (D4) days; they were imaged using EVOS® FL Cell Imaging System at 4× magnification (Scale bar 1,000 μm). The boundary lines were simply drawn using the shape tool from PowerPoint. (**H**) JICD overexpression does not affect the weights of organs (spleen, liver, prostate gland, testis, and kidney). Data are shown as mean ± SEM of two independent biological *in vivo* experiments (n = 6 mice per each group). ns, not significant; two-tailed t-test.

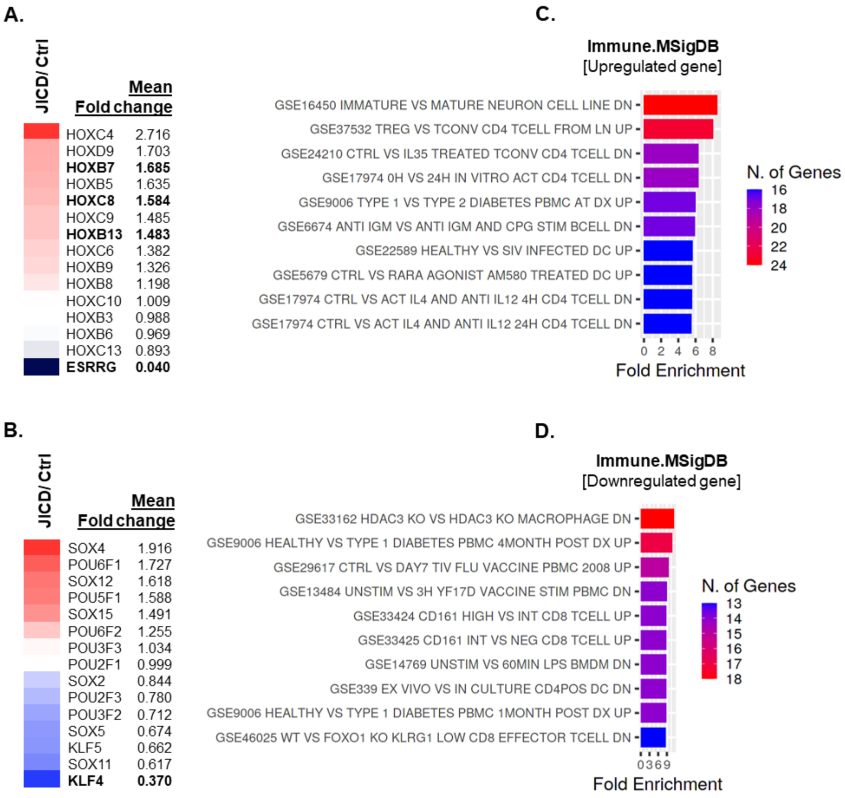

Figure S6. JICD overexpression alters the expression of genes facilitating CRPC progression. (**A-B**) JICD overexpression enhances CRPC gene signatures. Heatmap showing the depletion of ERRɣ and the upregulation of homeobox (HOX) gene family in JICD-overexpressing CWR22Rv1 cells (**A**) and changes in the expression level of embryonic stem cell (ESC)-related genes (**B**). (**C-D**) JICD alters immune gene signatures. Gene set enrichment analysis using the curated immune Molecular Signature database (Immune.MSigDB) and showing the upregulation of genes involved in immune tolerance (**C**) and the downregulation of genes involved in immune defense (**D**) in JICD-overexpressing CWR22Rv1 cells. False discovery rate (FDR) < 0.05.

WB (Fig. 1A)

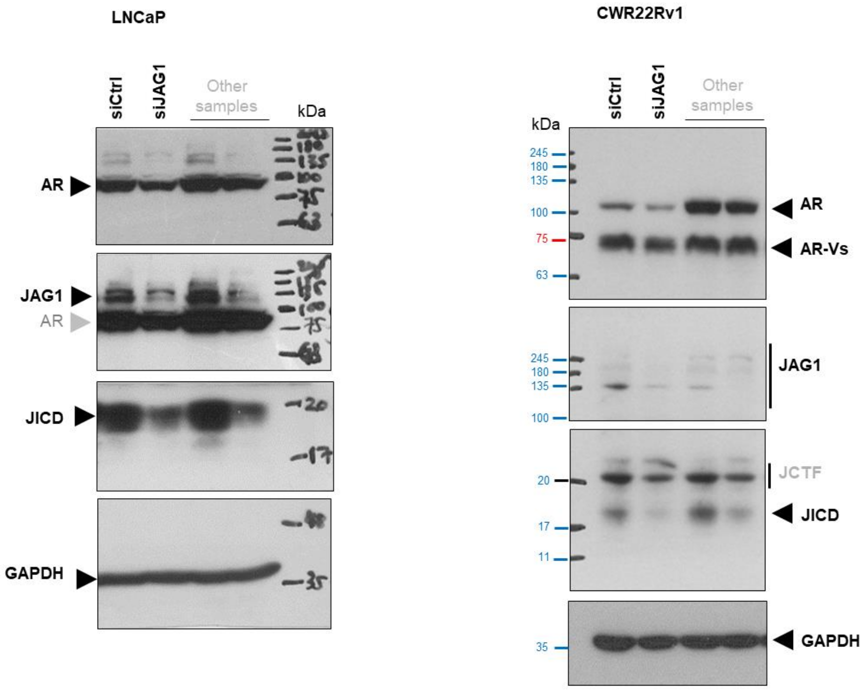
LNCaP
siCtrl
siJAG1
Other samples
kDa
AR
JAG1
AR
JICD
GAPDH
CWR22Rv1
siCtrl
siJAG1
Other samples
kDa
245
180
135
100
75
63
AR
AR-Vs
245
180
135
100
JAG1
20
17
11
JCTF
JICD
35
GAPDH

Gel (Fig. 1B, left)

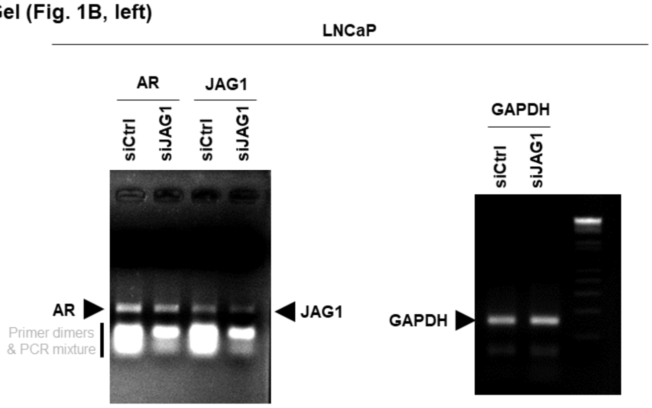

Gel (Fig. 1B, right)

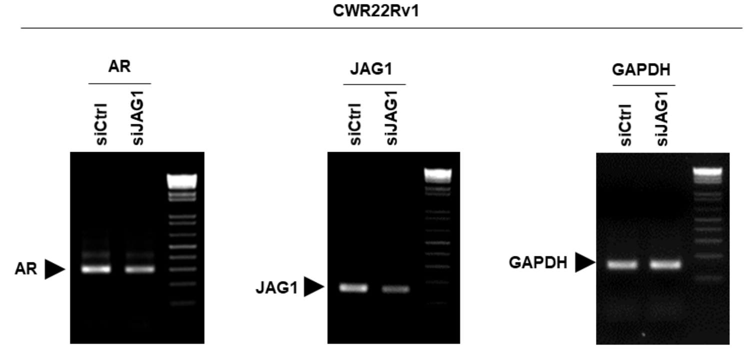

WB (Fig. 1C)

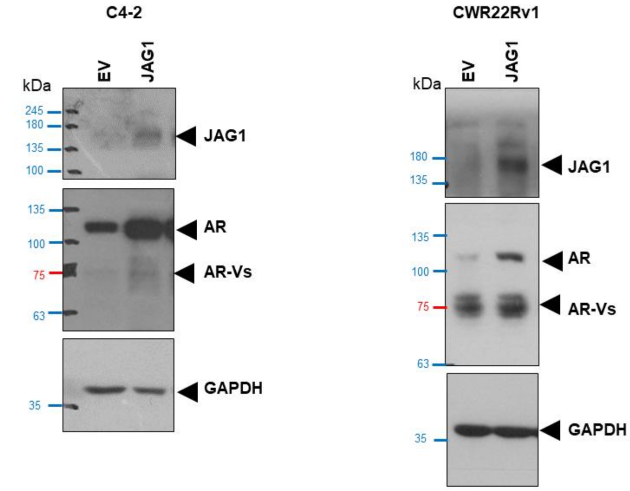

Gel (Fig. 1D, left)

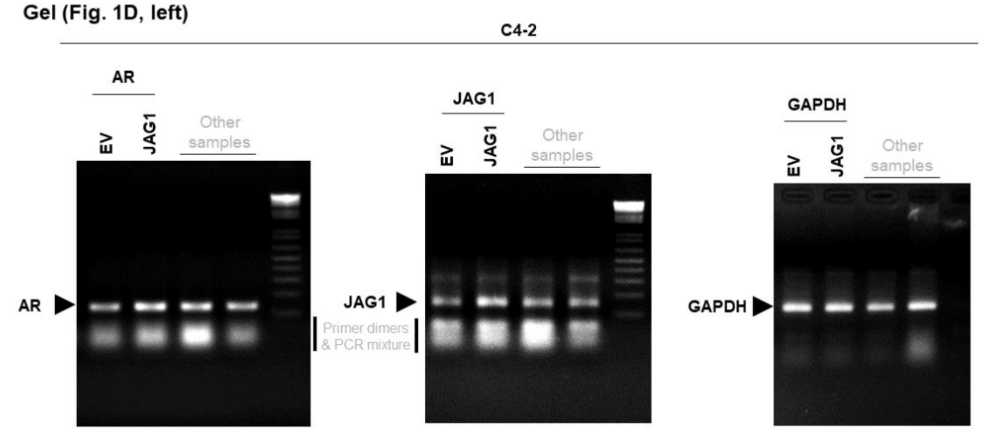

Gel (Fig. 1D, right)

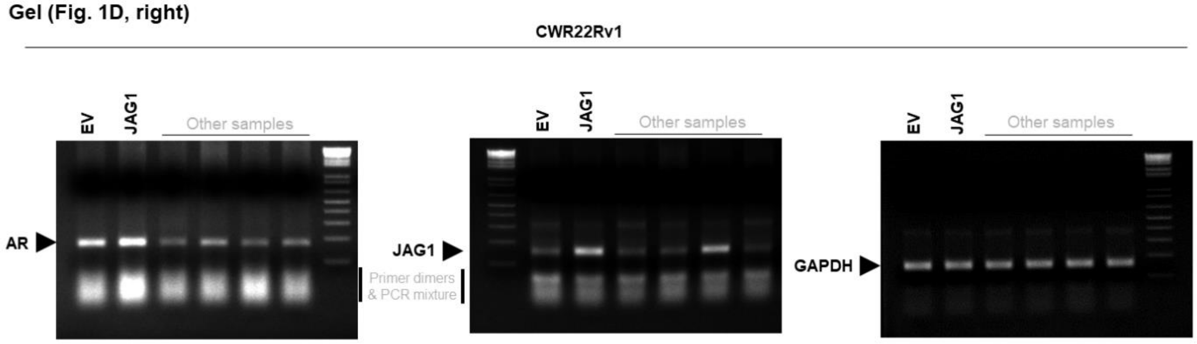

WB (Fig. 2B)

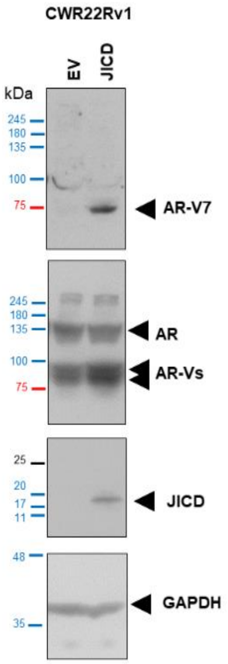

WB (Fig. 2A)

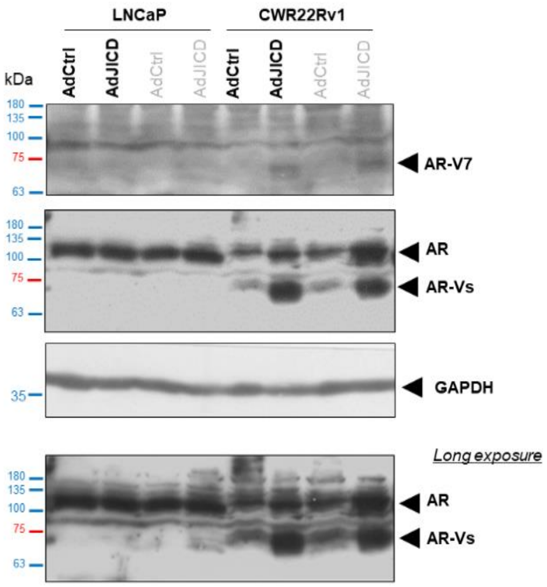

Gel (Fig. 2C)

CWR22Rv1

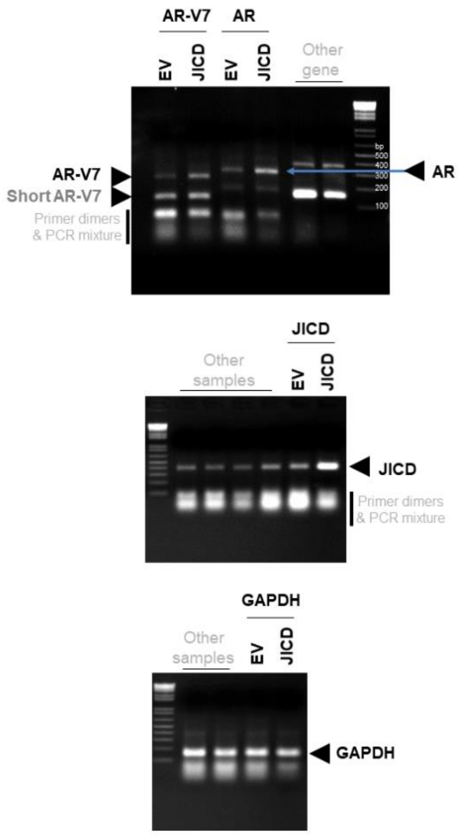

WB (Fig. 4A)

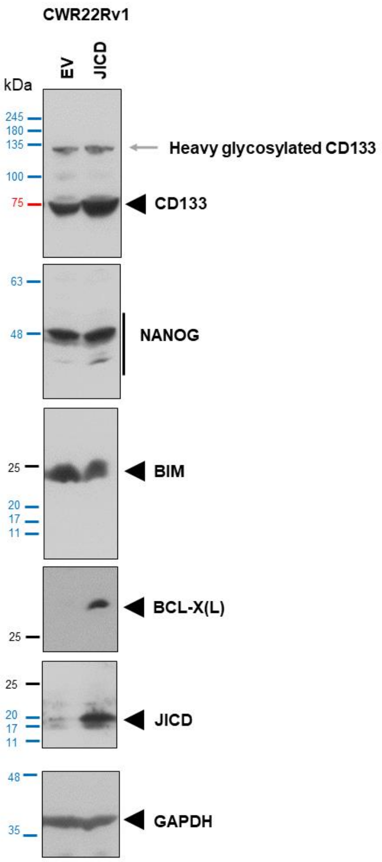

Gel (Fig. 4B)

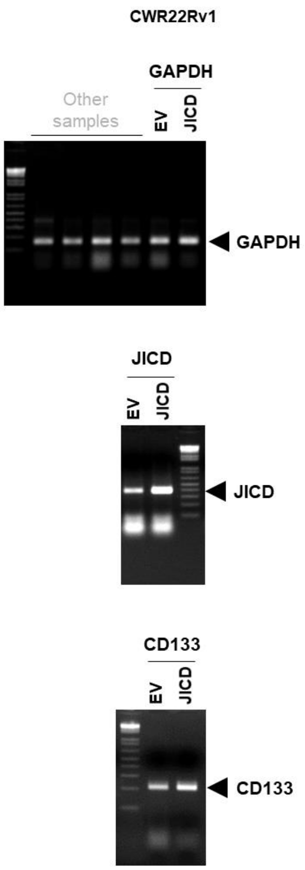

WB (Fig. 4F)

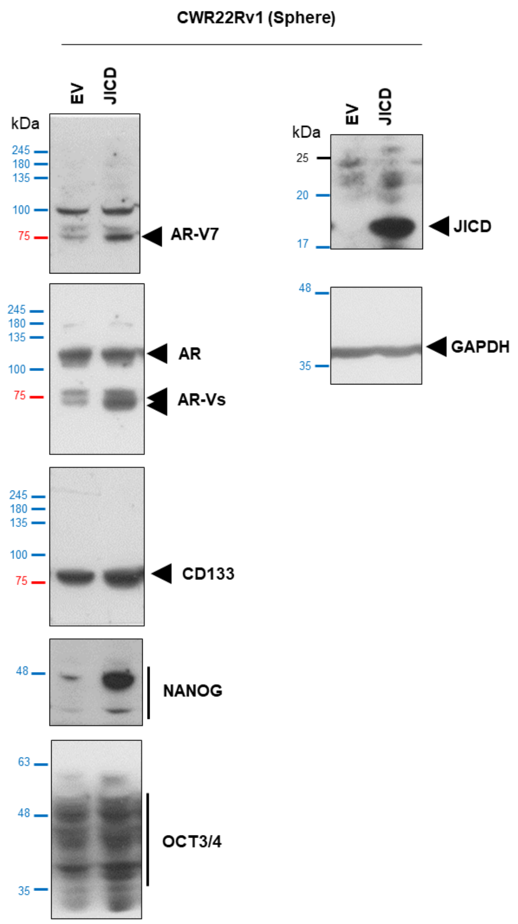
CWR22Rv1 (Sphere)
EV
JICD
kDa
245
180
135
100
75
AR-V7
AR
AR-Vs
CD133
48
NANOG
63
35
OCT3/4
25
20
17
JICD
GAPDH

WB (Fig. S1)

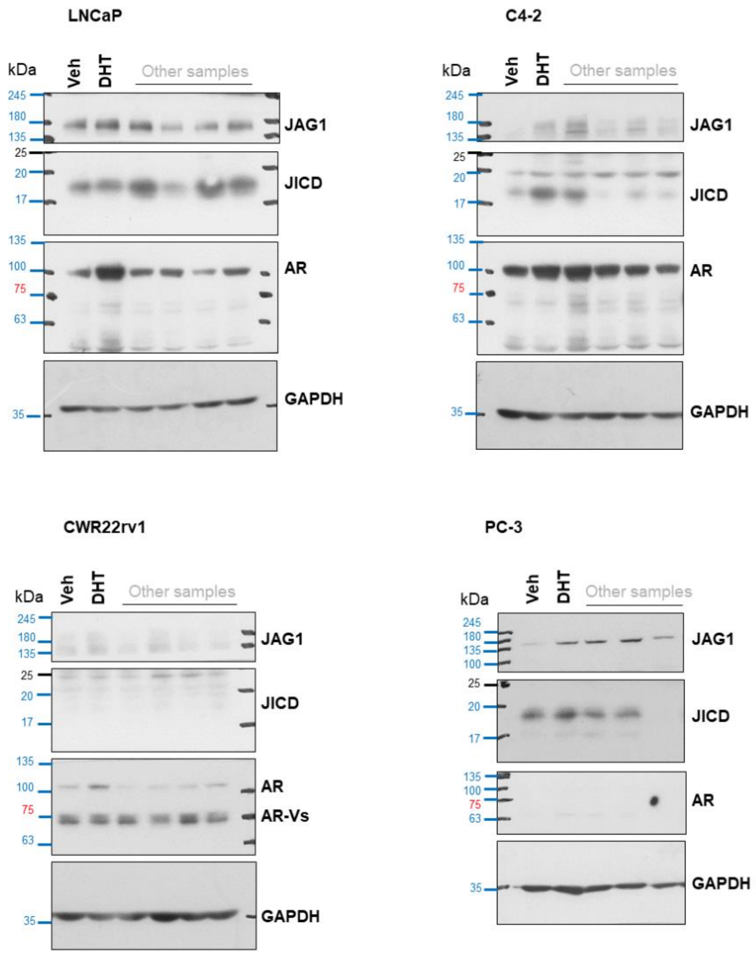

WB (Fig. S3)

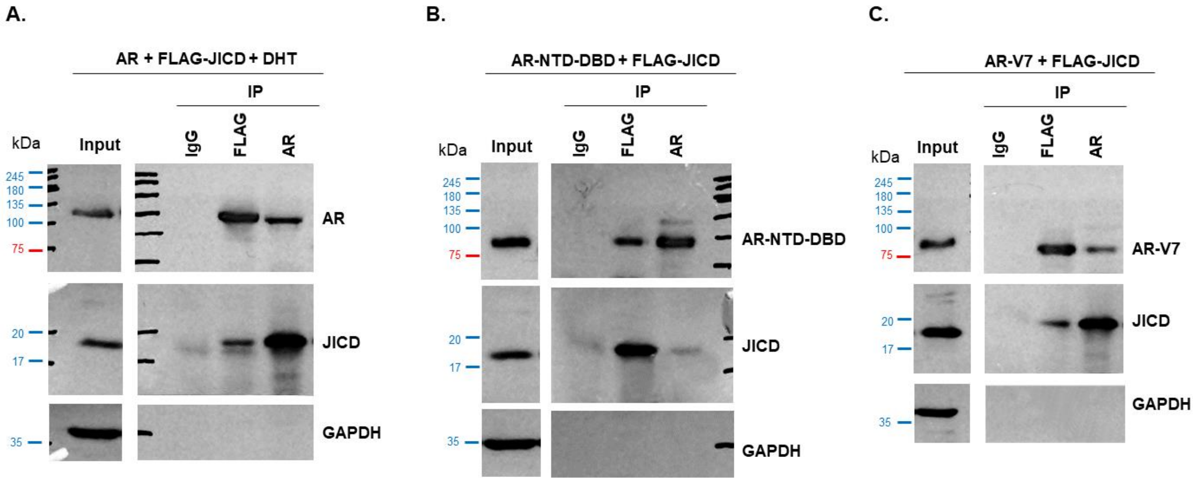

WB (Fig. S4A)

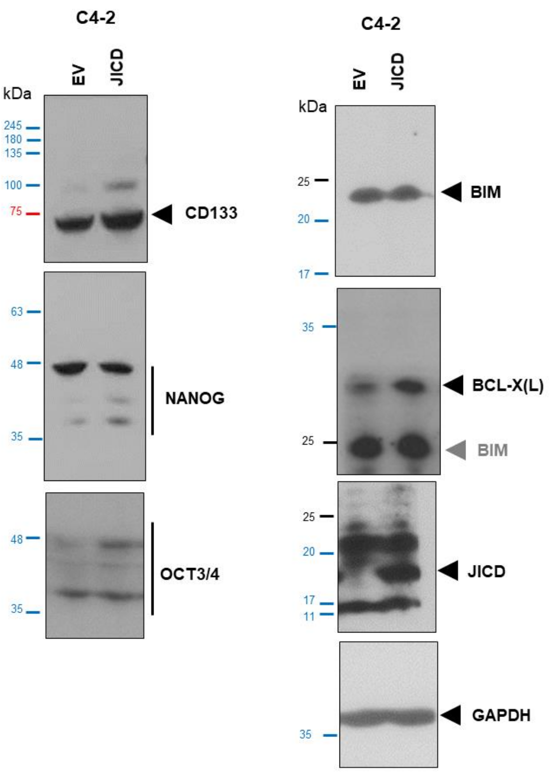

Gel (Fig. S4B)

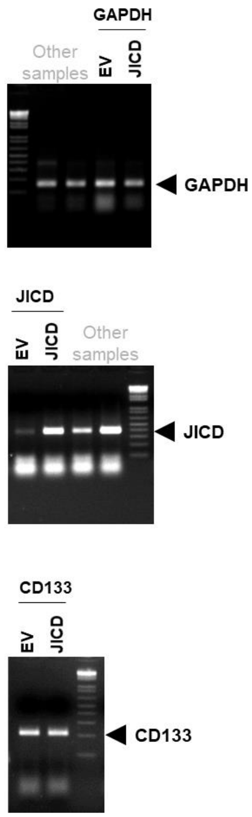

Gel (Fig. S4C)

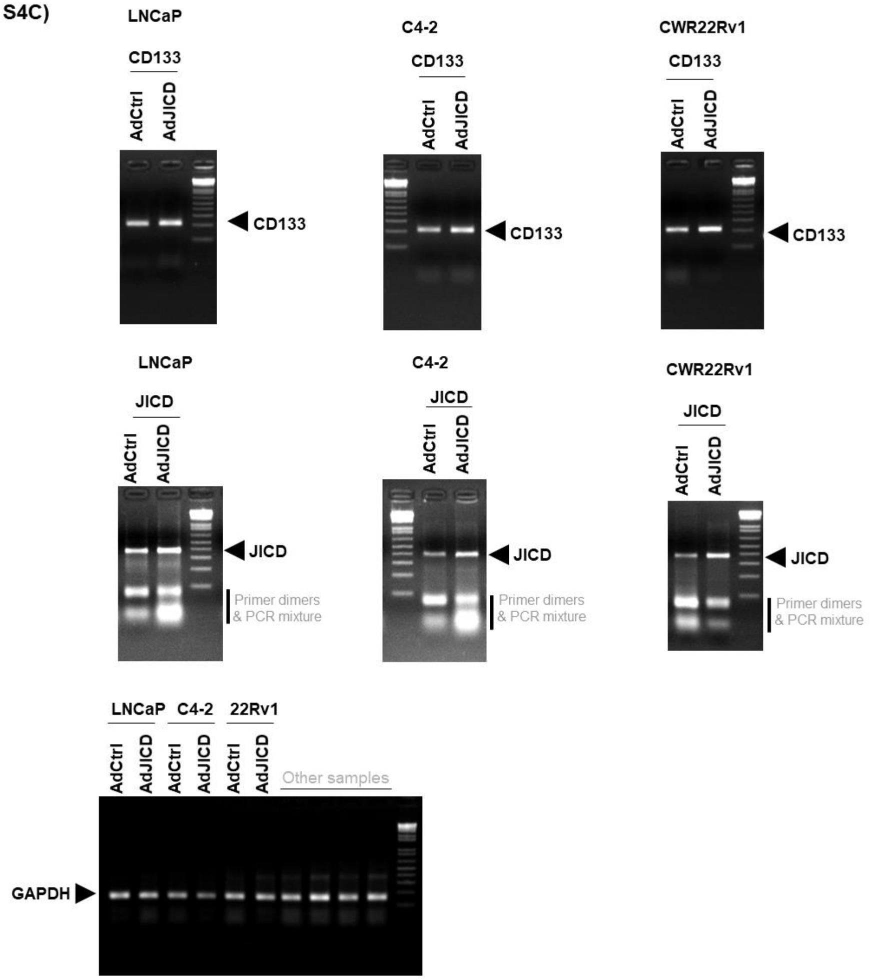
LNCaP
CD133
AdCtrl
AdJICD
CD133
C4-2
CD133
AdCtrl
AdJICD
CD133
CWR22Rv1
CD133
AdCtrl
AdJICD
CD133
LNCaP
JICD
AdCtrl
AdJICD
JICD
Primer dimers
& PCR mixture
C4-2
JICD
AdCtrl
AdJICD
JICD
Primer dimers
& PCR mixture
CWR22Rv1
JICD
AdCtrl
AdJICD
JICD
Primer dimers
& PCR mixture
LNCaP
C4-2
22Rv1
AdCtrl
AdJICD
AdCtrl
AdJICD
AdCtrl
AdJICD
Other samples
GAPDH

WB (Fig. S4D)

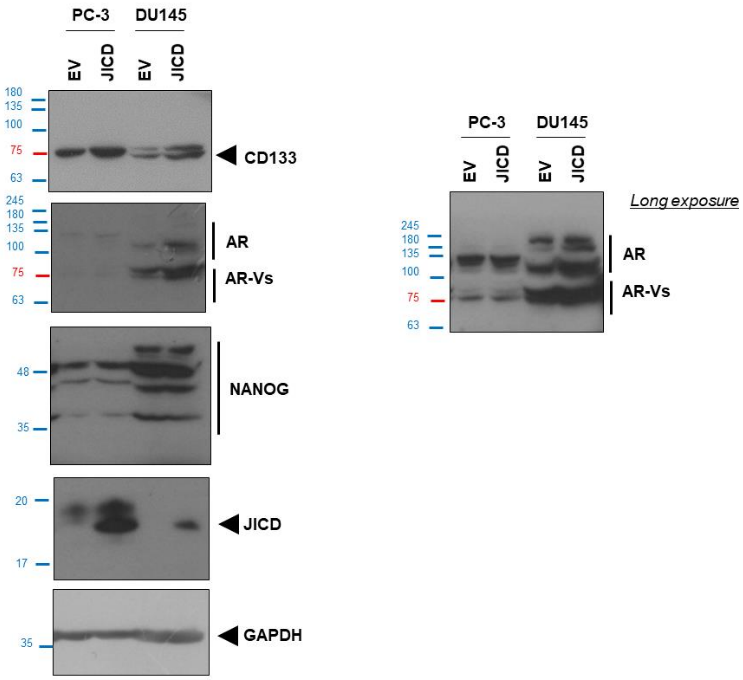

PC-3
DU145
EV
JICD
EV
JICD
180
135
100
75
63
CD133
245
180
135
100
75
63
AR
AR-Vs
48
35
NANOG
20
17
JICD
35
GAPDH
Long exposure
PC-3
DU145
EV
JICD
EV
JICD
245
180
135
100
75
63
AR
AR-Vs

Gel (Fig. S5A)

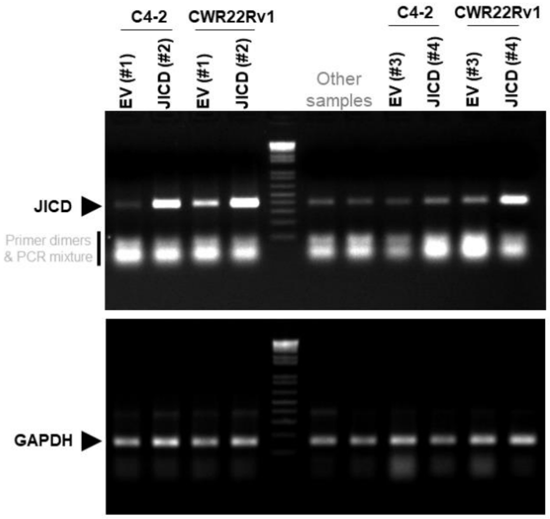

Figure S7. Original uncropped western blot and gel images (Supporting data).

3. Supplementary tables

Table S1. Oligonucleotides.

Cloning	JICD-Forward: ATAGAATTCCACACACACTCAGCCTCTG
	JICD-Reverse: CGTGATATCCTATACGATGTACTCCATTCG
RT-PCR	Total AR e1-Forward: GAAATGGGCCCCTGGATG
	Total AR e2-Reverse: CATCTCCACAGATCAGGCAGG
	JAG1 Forward: ACCCGATCAAGGAAATCACTG
	JAG1 Reverse: GAGCTCAGCAAGGGAACAAG
	e3-AR Forward: TGCACTATTGATAAATTCCGAAGG
	CE1-AR Reverse: CAAACACCCTCAAGATTCTTTCAG
	CE3-AR Reverse: GTCATTTTGAGATGCTTGCAATTG
	e4-AR Reverse: TTCTGGGTTGTCTCCTCAGT
	JICD-Forward: ATAGAATTCCACACACACTCAGCCTCTG
	JICD-Reverse: CGTGATATCCTATACGATGTACTCCATTCG
	CD133 Forward: ACATGAAAAGACCTGGGGG
	CD133 Reverse: GATCTGGTGTCCCAGCATG
	Oct3/4 Forward: AGTCGGGGTGGAGAGCAACT
	Oct3/4 Reverse: CCAGGGTGATCCTCTTCTGCTTC
	GAPDH Forward: ATCACCATCTTCCAGGAGCGAG
	GAPDH Reverse: GAGATGATGACCCTTTTGGCTCC
siRNA oligo	siJAG1: CCUGUAACAUAGCCCGAAA(dTdT)
	siCtrl: ACCCCGGAGAUGCUACCCGAA(dTdT)

Table S2. Antibodies and sources.

**Antibodies**	**Sources**
Anti-NANOG (ab155943)	Abcam
Anti-AR-V7 (68492); anti-CD133 (5860)	Cell Signaling
Anti-BCl-XL (1018-1), anti-BIM (1036-1)	Epitomics
Anti-AR (06-680)	Millipore
Anti-AR (sc-7305), anti-GAPDH (sc-25778), anti-JAG1 (sc-8303); anti-OCT3/4 (sc-5079)	Santa Cruz
Anti-FLAG (F7425)	Sigma-Aldrich
Goat anti-mouse IgG (H+L), HRP (31430); goat anti-rabbit IgG (H+L), HRP (31460)	ThermoFisher Scientific

Supplementary reference

1. Palumbo-Zerr, K.; Zerr, P.; Distler, A.; Fliehr, J.; Mancuso, R.; Huang, J.; Mielenz, D.; Tomcik, M.; Furnrohr, B.G.; Scholtysek, C.; et al. Orphan nuclear receptor NR4A1 regulates transforming growth factor-beta signaling and fibrosis. *Nat Med* **2015**, *21*, 150-158, doi:10.1038/nm.3777.
2. Malik, R.; Khan, A.P.; Asangani, I.A.; Cieslik, M.; Prensner, J.R.; Wang, X.; Iyer, M.K.; Jiang, X.; Borkin, D.; Escara-Wilke, J.; et al. Targeting the MLL complex in castration-resistant prostate cancer. *Nat Med* **2015**, *21*, 344-352, doi:10.1038/nm.3830.
